# Optical and Chiroptical Stimuli-Responsive Chiral AgNPs@H-Leu-Poly(phenylacetylene) Nanocomposites in Water

**DOI:** 10.1021/acsnano.4c08622

**Published:** 2024-10-09

**Authors:** Manuel Fernández-Míguez, Manuel Núñez-Martínez, Esteban Suárez-Picado, Emilio Quiñoá, Félix Freire

**Affiliations:** Centro Singular de Investigación en Química Biolóxica e Materiais Moleculares (CiQUS) and Departamento de Química Orgánica, Universidade de Santiago de Compostela, 15782 Santiago de Compostela, Spain

**Keywords:** chirality, silver nanoparticles, stimuli responsiveness, poly(phenylacetylene)s, nanocomposites

## Abstract

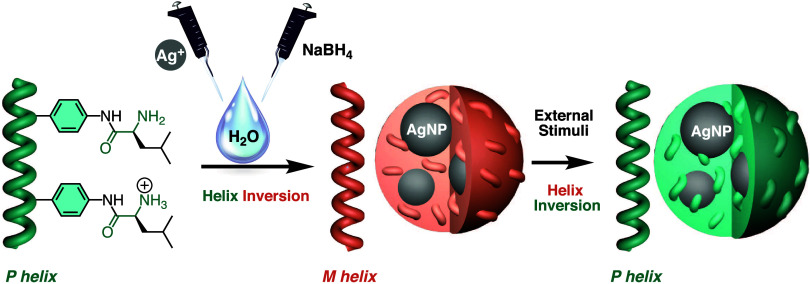

Dynamic macroscopically chiral nanocomposites are prepared by combining silver nanoparticles (AgNPs) and dynamic helical poly(phenylacetylene)s (PPAs) bearing pendants functionalized with amino groups. These amino groups provide the nanocomposite with the ability to disperse in water along with high stability due to the interaction between the ammonium group and the AgNP. Moreover, the equilibrium between NH_3_^+^/NH_2_ produces a “blinking” contact between the PPA and the AgNPs, which allows total control of the dynamic helical behavior of the polymer. The use of acidic or neutral pH allows controlling the morphology of the nanocomposite, which consists of a nanosphere that has trapped inside it a single AgNP (pH = 2) or several AgNPs (pH = 7) with ca. 30 nm of diameter. These nanocomposites combine the optical and chiroptical stimuli-responsive properties of both components, AgNPs and PPAs. Thus, the controlled aggregation of the nanocomposite produced variations in the LSPR band of the AgNPs in a reversible manner. In turn, given that the chiral coating is selective to Ba^2+^, the presence of this metal ion caused a helical inversion of the chiral coating of the nanocomposite detected by electronic circular dichroism. Moreover, it is possible to distinguish between three metal ions in different oxidation states, such as Ce^4+^, Fe^3+^, and Hg^2+^, which produce different responses of the nanocomposite when oxidizing the AgNP to Ag^+^.

## Introduction

Silver nanoparticles (AgNPs) have attracted the attention of the scientific community due to their applications in various fields such as medical therapy,^1^ sensors,^2^ and catalysis,^3^ among others. However, to keep these AgNPs stable over time in specific environmental conditions, it is necessary to coat them with a certain organic molecule or polymeric coating.^4−7^ Thus, their natural tendency to aggregate and oxidize is disrupted by steric or electrostatic effects.^8^ Moreover, by playing with the structure and functional groups of the coating agents, extra functionalities can be introduced in the hybrid material that could combine the properties of both systems (MNPs and coating)^9^ generating chiral plasmonic nanostructures.^10−13^

Helical biopolymers including DNA,^14^ peptides,^15^ and polysaccharides^16^ have been used to decorate AgNPs. However, their pool of building blocks (chirality, functional groups) in biopolymers is limited because Nature only used a limited number of molecules to create organisms, such as the 20 natural amino acids or the 4 nucleotides. Furthermore, these biomacromolecules are generally static, a fact that makes it not possible to tune their helical sense or elongation in the presence of external stimuli.

Our group has been working lately on the preparation of hybrid materials that combine the properties of MNPs (M = Au or Ag) and stimuli-responsive helical polymers such as poly(phenylacetylene)s (PPAs).^17−20^ These polymers are dynamic from a structural point of view, a fact that allows their secondary structure (elongation and/or helical sense, scaffold) to be modulated by the presence of external stimuli such as temperature, solvents, metal ions and pH, among others.^21−31^ These characteristics make them excellent candidates to protect MNPs and obtain hybrid materials (MNPs@PPAs) that combine the dynamic chiroptical properties of PPAs and the optical properties of MNPs.

However, to date, these studies were carried out only in organic media (e.g., CHCl_3_, DCM), due to the limitation of creating water-soluble PPAs with the ability to interact with MNPs. The main reason is that the Rh(I) catalyst used during the polymerization reaction is poisoned by monomers containing amino groups. This problem was recently overcome by performing the polymerization reaction with protonated ammonium phenylacetylene monomers (NH_3_^+^-PA).^32^ In this acidic conditions, the Rh(I) catalyst is not poisoned, allowing the creation of water-soluble amino-PPAs required to prepare water-soluble MNPs@PPA nanocomposites. Furthermore, by using amino-PPAs and playing with the pH, it is possible to adjust the NH_3_^+^/NH_2_ ratio along the helical polymer. These amino and ammonium groups are in equilibrium and the positive charges, from a macroscopic point of view, exchange from one amino group to another along the polymer scaffold.^33−35^ Therefore, the NH_3_^+^—AgNP contacts, which stabilize the metal nanoparticle, are dynamic and move along the polymer–metal nanoparticle interface, without blocking the flexibility of the pendant groups attached to the AgNP. As a result, the polymer is expected to vary its helical structure—elongation and helix sense—when it interacts with external stimuli in the nanocomposite, similarly to its behavior in the molecularly dissolved state.

**Scheme 1:**
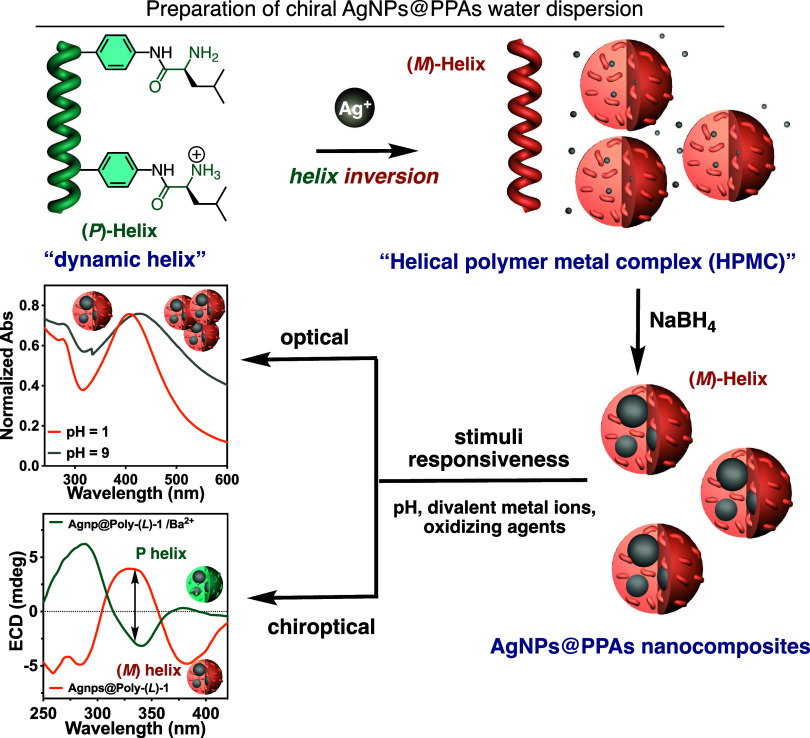
Graphical Illustration for the Preparation AgNPs@PPAs Nanocomposites Dispersed in Water and the Stimuli-Responsive Properties Addressed in This Work

In this work, we will explore the formation, chiroptical properties, and morphology of dynamic aqueous AgNPs@PPAs nanocomposites by reducing an amino-PPA/Ag^+^ complex at acidic and neutral pH (2 and 7 respectively, Scheme 1) and their stimuli-responsive properties in water against different stimuli such as pH, divalent metal ions, and oxidizing agents.

## Results and Discussion

**Figure 1:**
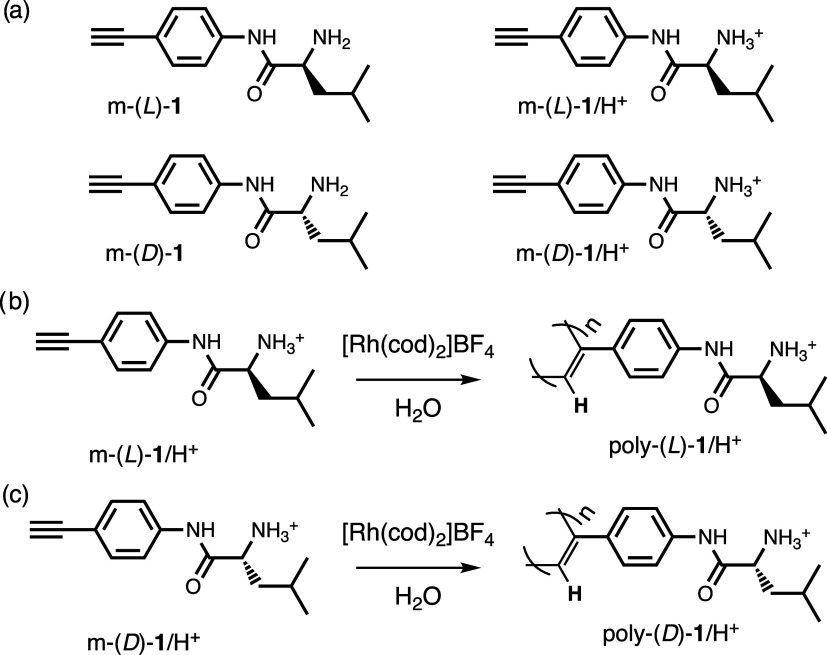
Chemical structure of (a) m-(*L*)-**1**, m-(*D*)-**1**, and (b) their protonated forms. (c) Scheme of the polymerization reaction to obtain Poly(*L*)-**1**/H^+^ and Poly(*L*)-**1**/H^+^.

To prepare a chiral AgNPs@PPA nanocomposite that can be dispersed in water, we designed an aminophenylacetylene monomer bearing the 4-ethynyl anilide of (*L*)- or (*D*)-leucine with a deprotected amino group (Figure 1a). These monomers were prepared following peptide chemistry (Supporting Information) and were further protonated with HCl (1 equiv) to generate the corresponding ammonium salt (Figure 1b).

**Figure 2:**
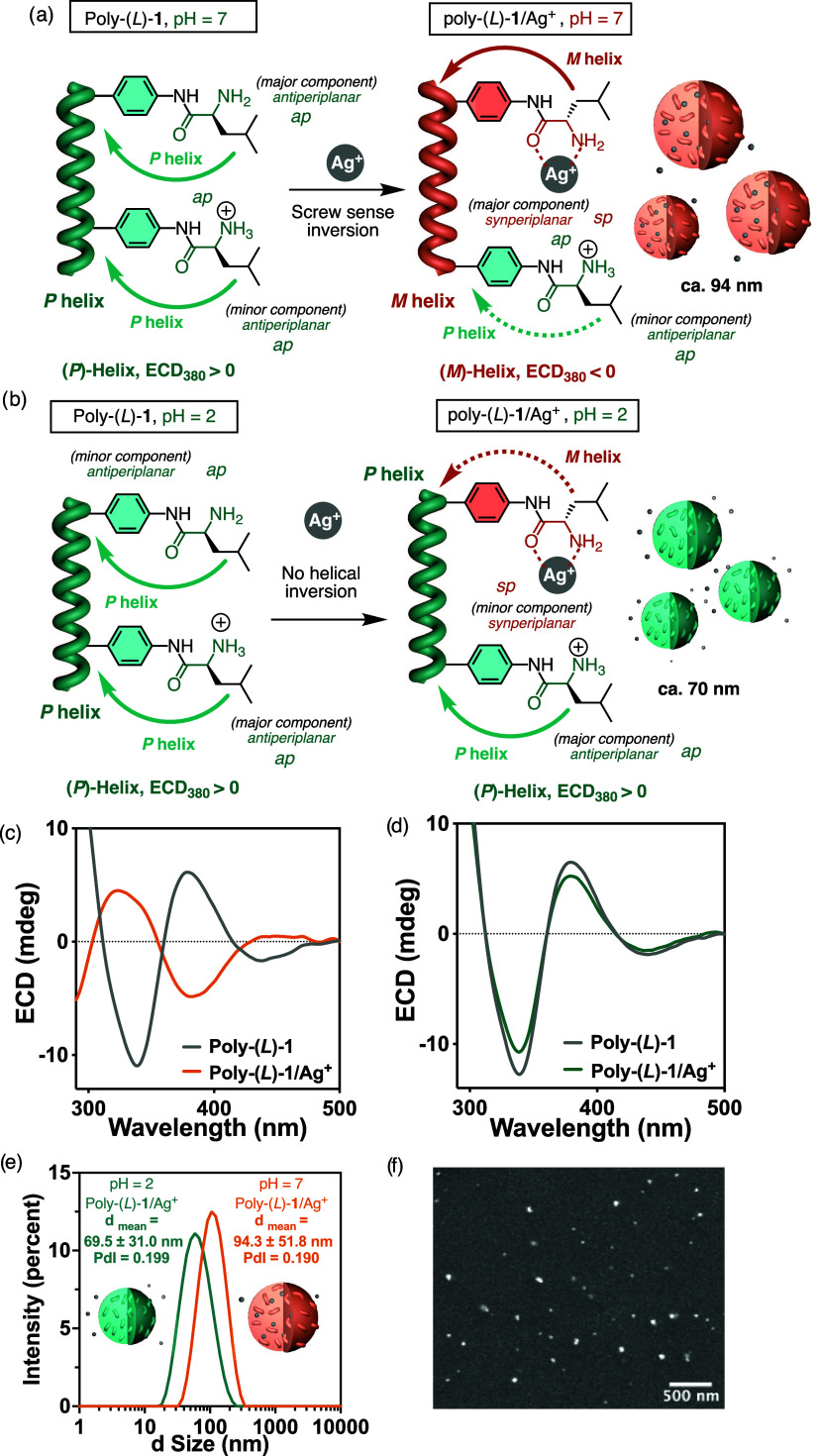
(a) Schematic illustration of the Poly(*L*)-**1** helix inversion after complexation with 1.5 equiv of Ag^+^ at pH = 7 and (b) pH = 2 and the formation of HPMC particles. ECD spectra of Poly(*L*)-**1** and Poly(*L*)-**1**/Ag^+^ at (c) pH = 7 and (d) pH = 2. (e) DLS studies of Poly(*L*)-**1**/Ag^+^ 1/1.5 mol/mol prepared at pH = 7 and pH = 2. (f) SEM image of Poly(*L*)-**1**/Ag^+^ nanospheres at pH = 7. [Poly(*L*)-**1**] = 0.3 mg/mL H_2_O, [AgClO_4_] = 10 mg/mL H_2_O.

These protonated monomers were polymerized with [Rh(cod)_2_]BF_4_ in water to obtain the desired polymers (Poly(*L*)-**1**/HCl and Poly(*D*)-**1**/HCl) with low polydispersity and high content of *cis-*configuration of double bonds (Figure 1c). These polymers show good water solubility at different pH, e.g., pH = 2 and 7, where the NH_2_/NH_3_^+^ ratio will be different along the polymeric scaffolds. Both protonated and deprotonated forms of the amino group are needed to prepare the desired chiral AgNPs@PPA nanocomposite, with the NH_2_ groups being necessary to form complexes with Ag^+^ ions, while the ammonium groups are essential to providing water solubility. Under these conditions (pH = 2 and pH = 7), Poly(*L*)-**1** shows a *P* screw sense excess inferred from ECD (ECD_380 nm_> 0), while Poly(*D*)-**1** shows an *M* screw sense excess (ECD_380 nm_< 0) due to their enantiomeric relationship (Figures 2c,d and S10). The adoption of a *P* helix by Poly(*L*)-**1** and an *M* helix by Poly(*D*)-**1** is due to the presence of a preferred *antiperiplanar* conformation between the carbonyl and NH_2_/NH_3_^+^ groups in the pendant (Figure 2a,b), which places the side chain of the amino acid, i.e., the isobutyl group, in such a way that it induces a *P* helix in the main chain of the polyene (Figure 2a).

Next, the ability of Poly(*L*)-**1** to form complexes with Ag^+^ ions at different pHs was verified by adding AgClO_4_ (1.5 equiv, *c* = 10 mg/mL MQ H_2_O) to an aqueous solution of Poly(*L*)-**1** at pH = 7 and 2 (Figure 2a,b). Interestingly, when complexation is carried out at pH = 7 (Figure 2a), a helix inversion from *P* (ECD_380 nm_> 0) to *M* (ECD_380 nm_< 0) is observed (Figure 2c). This is a consequence of a change in conformation in the pendant group from an *antiperiplanar* orientation between the carbonyl and amino (NH_2_) groups to a *synperiplanar* orientation after chelation of both functional groups with a silver(I) ion (Figure 2a). This *antiperiplanar* to *synperiplanar* conformational switch places the amino acid side chain, i.e., the isobutyl group, in two very different spatial orientations, producing a helix inversion effect.

However, at pH = 2, the addition of AgClO_4_ to a Poly(*L*)-**1** solution produces a slight decrease in the *P* screw sense excess (Figure 2b,d). This result can be explained by considering the ratio of NH_2_/NH_3_^+^ groups within Poly(*L*)-**1** at these pHs. Thus, while at pH = 7, the amount of neutral amino groups, which can coordinate silver ions, is high (major component); at acidic pH (pH = 2), this number is very low (minor component), the helix being mostly populated by ammonium groups that have lost the ability to coordinate with Ag^+^ ions. Interestingly, these helical polymer metal complexes (HPMC, Poly(*L*)-**1**/Ag^+^), prepared at pH = 7 and pH = 2, form nanospheres in water as inferred from dynamic light scattering (DLS) (Figure 2e) and scanning electron microscopy (SEM) studies (Figures 2f and S23–S26).

HPMC nanospheres are generated due to the ability of silver ions to act as cross-linking agents. Their size is larger at pH = 7 due to the greater number of Ag^+^ that form complexes with the amino groups (NH_2_) of different polymer chains, which are more abundant at pH = 7 than at pH = 2.

Analogous studies were carried out with Poly(*D*)-**1**, showing identical stimuli-responsive properties but opposite helical senses due to their enantiomeric relationship (Figure S10).

**Figure 3:**
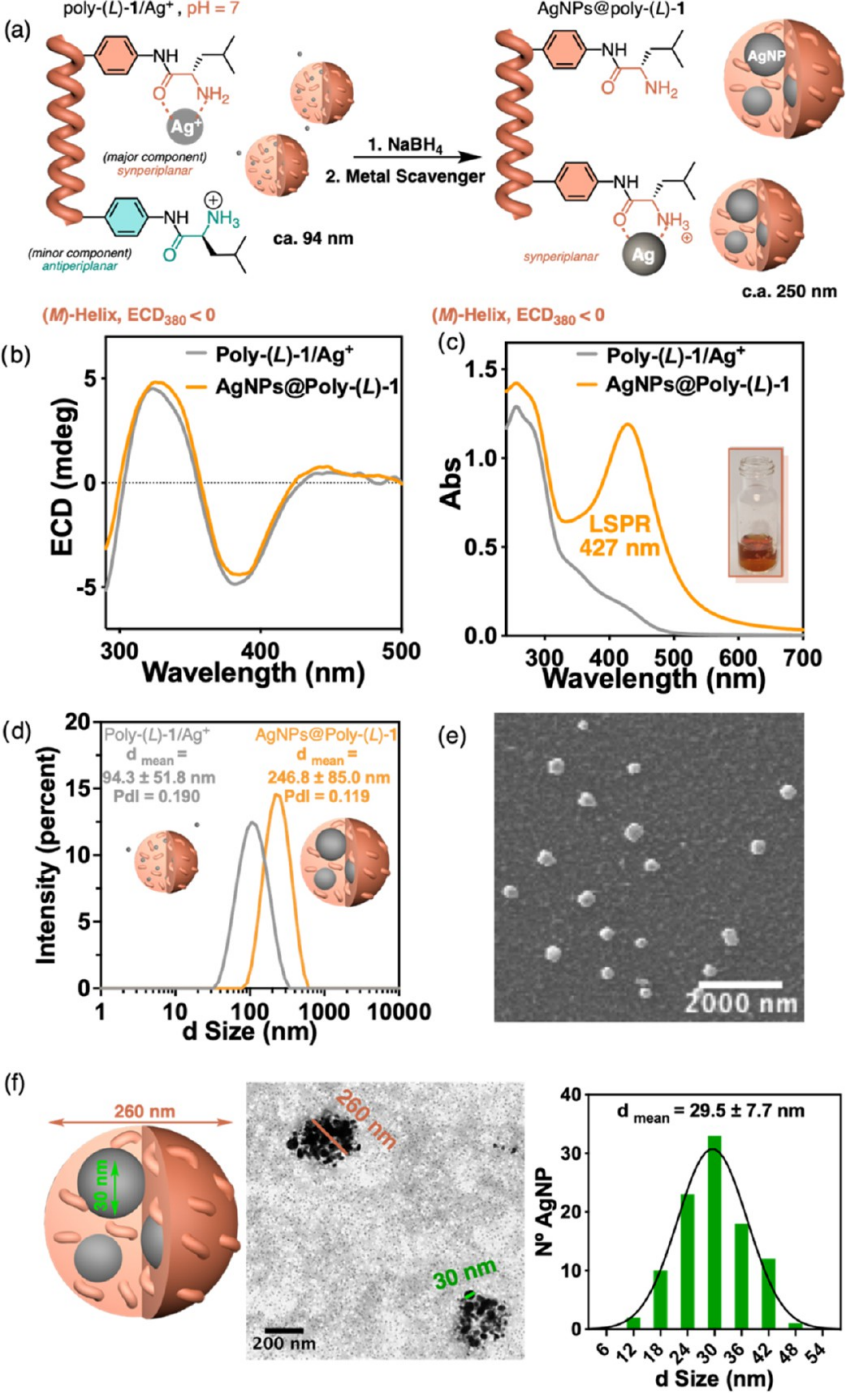
(a) Schematic illustration of the formation of AgNPs@Poly(*L*)-**1** nanocomposite at pH = 7. Comparison of (b) ECD and (c) UV–vis spectra of Poly(*L*)-**1**/Ag^+^ and AgNPs@Poly(*L*)-1 at pH = 7. (d) DLS studies of Poly(*L*)-**1**/Ag^+^ and AgNPs@Poly(*L*)-**1**. (e) SEM and (f) TEM studies of AgNPs@Poly(*L*)-**1** at pH = 7 (Gaussian size distribution of 100 AgNPs).

**Figure 4:**
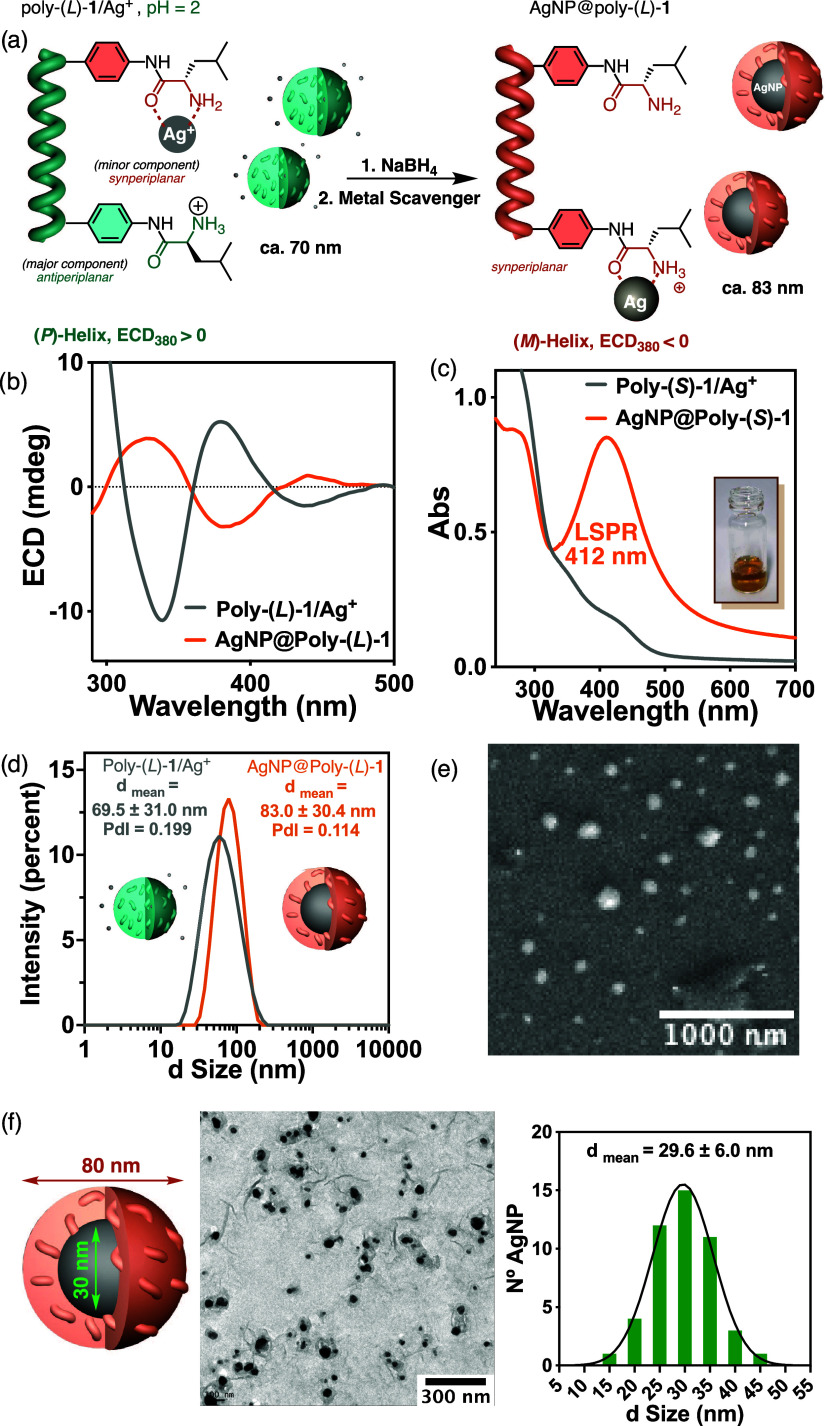
(a) Schematic illustration of the formation of AgNP@Poly(*L*)-**1** nanocomposite at pH = 2. Comparison of (b) ECD and (c) UV–vis spectra of Poly(*L*)-**1**/Ag^+^ and AgNP@Poly(*L*)-**1** at pH = 2. (d) DLS studies of Poly(*L*)-**1**/Ag^+^ and AgNP@Poly(*L*)-**1**. (e) SEM and (f) TEM studies of AgNP@Poly(*L*)-**1** at pH = 2 (Gaussian size distribution of 50 AgNPs).

Next, AgNPs@Poly(*L*)-**1** nanocomposites were prepared at pH = 7 (Figure 3a) and pH = 2 (Figure 4a) from the Poly(*L*)-**1**/Ag^+^ complexes (1/1 mol/mol ratio) by adding 0.45 equiv of NaBH_4_ as the reducing agent. The mixtures were kept under vigorous stirring for 60 min, and the color of the solutions changed from yellow to brown, indicative that reduction of silver ions was taking place. Immediately thereafter, a metal scavenger resin (Quadrapure TU) was added to the solution mixtures to remove unreacted Ag^+^ ions and Na^+^ from the reducing agent.

ECD studies at pH = 7 show that the formation of the AgNPs@Poly(*L*)-**1** nanocomposite, after metal ion reduction, does not affect the preferred *P* screw sense adopted by Poly(*L*)-**1**/Ag^+^—ECD_380 nm_< 0 for Poly(*L*)-**1**/Ag^+^ and AgNPs@Poly(*L*)-**1** (Figure 3b). UV–vis studies of AgNPs@Poly(*L*)-**1** show the localized surface plasmon resonance (LSPR) band centered at 427 nm, indicative of AgNPs formation (Figure 3c). Moreover, a variation in the size of the aggregate during the formation of the composite, from 94 to ca. 250 nm, is observed by DLS and SEM. These studies also reveal the presence of spherical particles for the AgNPs@Poly(*L*)-**1** nanocomposite (Figures 3d,e and S30). These nanospheres are filled with several AgNPs whose size is ca. 30 nm (Figure 3f), as could be observed in the images obtained through transmission electron microscopy (TEM) studies. The large number of AgNPs within the nanosphere that forms AgNPs@Poly(*L*)-**1** is attributed to the presence of several nucleation points (amino groups complexed with silver ions: NH_2_—Ag^+^) along the helix at pH = 7. Considering the morphology of the helical polymer metal complex and the nanocomposite, it is necessary for the reducing agent to diffuse inside the Poly(*L*)-**1**/Ag^+^ nanosphere to create nucleation sites.

Moreover, FT-IR studies show that in addition to the ammonium groups, the AgNPs are also stabilized by the carbonyl group of the anilide (Figure S11). As a result, these nanocomposites show good thermal and temporal stability once the solvent is removed and are redispersed in water, which confirms that Poly(*L*)-**1** is a good protecting agent for obtaining AgNPs (Figure S12a).

In addition, photostability studies show that poly(*L*)-**1** is more stable to light irradiation when the nanocomposite is prepared at pH = 7 than when it is molecularly dissolved (see Figure S12). Similarly, VT-ECD studies show how, in a molecularly dissolved state, the screw sense excess is lost at 340 K, while in the nanocomposite, it remains unaltered at the same temperature (see Figure S12).

Interestingly, when the AgNPs@Poly(*L*)-**1** nanocomposite is prepared at pH = 2 (Figure 4a), ECD studies show a helical inversion of the Poly(*L*)-**1/**Ag^+^ complex after reduction of the silver ion to form the AgNP@Poly(*L*)-**1** nanocomposite (Figure 4b). At this pH, there are many ammonium groups in the polymer that do not interact with Ag^+^ ions (Figure 4a). However, once the metal ion is reduced to Ag(0), the ammonium groups have a high affinity for the metal in the ground state (Figure 4a). Thus, the chelation of carbonyl and ammonium to Ag(0) nanoparticles changes the preferred conformation of the pendant group, which evolves from *antiperiplanar* to *synperiplanar* during the reduction of the metal ion, placing the helix directing group (isobutyl) in different spatial orientations. As a result, this conformational switch produced a helix inversion of PPA that allowed us to monitor the reduction of Ag^+^ to Ag(0) by ECD (Figure 4b). Therefore, Poly(*L*)-**1** in addition to stabilizing the metal nanoparticles can be used to monitor the reduction of Ag(I) to Ag(0) in water.

UV–vis studies of AgNP@Poly(*L*)-**1** show the localized surface plasmon resonance (LSPR) band centered at 412 nm, indicative of the formation of AgNPs (Figure 4c). This plasmon band is hypsochromically shifted by 15 nm compared to the plasmon band of AgNP@Poly(*L*)-**1** obtained at pH = 7. DLS studies show a small variation in the aggregate size during the formation of the nanocomposite from 70 to 83 nm (Figure 4d), in agreement with electron microscopy studies, SEM and TEM, which show the presence of spherical particles for AgNP@Poly(*L*)-**1** whose average size is ca. 80 nm (Figures 4e,f and S25). Interestingly, TEM studies reveal that within each nanosphere of the AgNP@Poly(*L*)-**1** nanocomposite prepared at pH = 2, there is only one AgNP whose size is ca. 30 nm (Figure 4f).

Considering the different morphologies of the AgNP@Poly(*L*)-**1** nanocomposites prepared at pH = 7 and pH = 2, it is possible to explain the UV–vis data considering a different environment for the AgNPs in both nanocomposites. More precisely, a bathochromic shift is observed for the plasmon band of the nanocomposite prepared at pH = 7 with respect to the band observed at pH = 2, although the size of the AgNPs is ca. 30 nm in both cases.

This variation in the morphology of the AgNP@Poly(*L*)-**1** nanocomposites prepared at pH = 7 and pH = 2 is related to the different numbers of amino groups coordinated to the metal ion before adding the reducing agent. Thus, while at pH = 7, the number of amino groups within the polymer is greater than at pH = 2, the number of Ag^+^ coordinated to these amino groups is also greater. These amino/Ag^+^ interactions are the nucleation sites within the nanosphere, which produce more AgNPs growth sites within the nanocomposite at pH = 7 than at pH = 2. As a result, the morphology of the dispersed nanocomposites depends on the pH used to prepare them.

These nanocomposites prepared at pH = 2 show good thermal and temporal stability once the solvent is removed and redispersed again in water, confirming that Poly(*L*)-**1** is a good protective agent to obtain AgNPs (Figure S12b).

**Figure 5:**
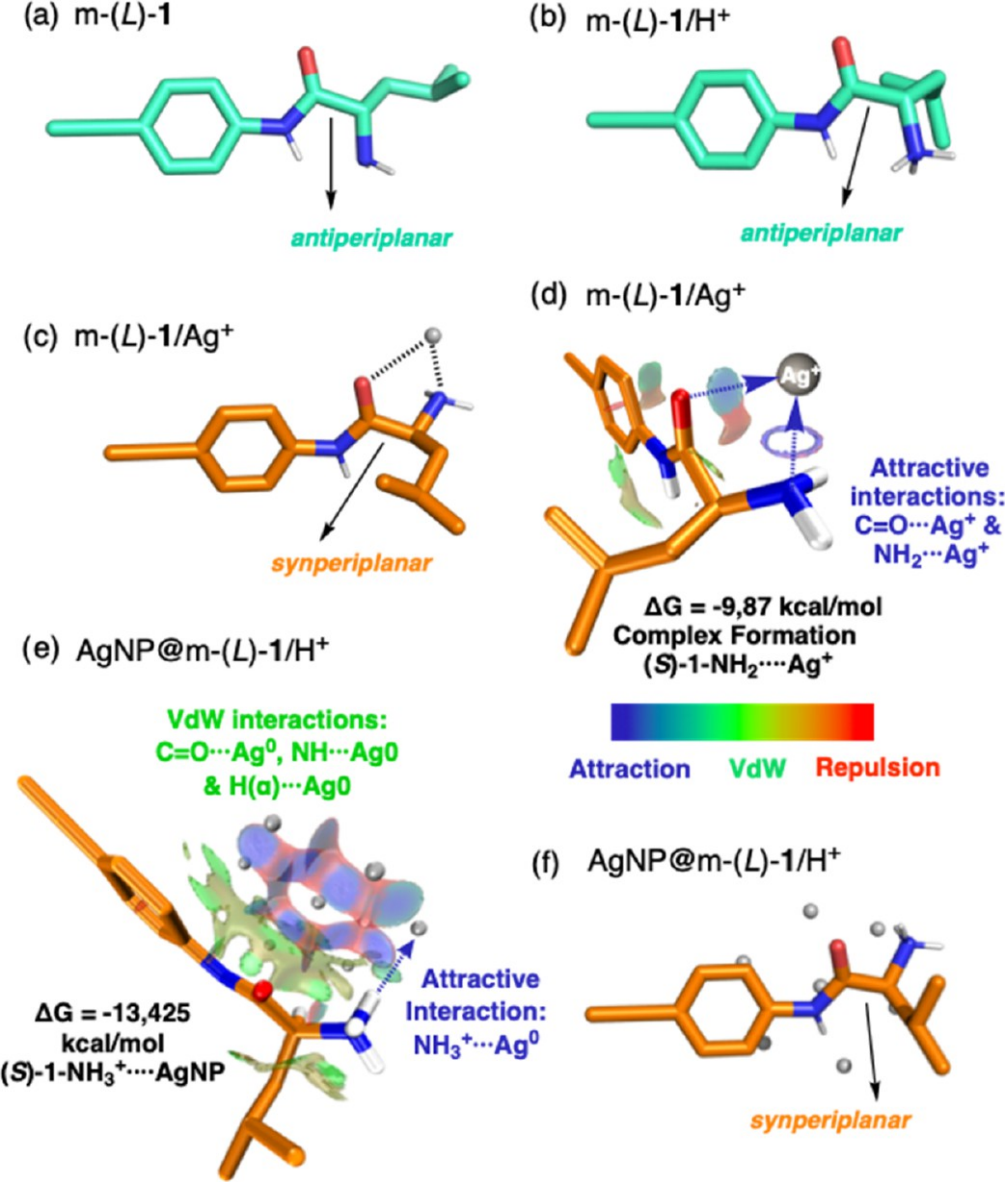
Preferred conformations of (a) m-(*L*)-**1** and (b) m-(*L*)-**1**/H^+^. (c) Preferred conformation of m-(*L*)-**1** in the presence of Ag^+^. (d) Graphical illustration showing the attractive noncovalent interactions of m-(*L*)-**1**/Ag^+^ obtained by NCI analysis. (e) Graphical illustration showing the attractive noncovalent interactions of m-(*L*)-**1**/H^+^/AgNP obtained by NCI analysis. (f) Preferred conformation of m-(*L*)-**1**/H^+^ in the presence of AgNPs. All structures were optimized by DFT B3LYP-D3/6-311+G(d,p) for all atoms and cc-pVDZ(-pp) for Ag.

Density functional theory calculations (SI) confirm the experimentally elucidated formation mechanism of AgNP(s)@Poly(*L*)-**1**. Thus, in the absence of any metal ion, DFT calculations [B3LYP-D3/6-311+G(d,p)] indicate that the preferred conformation in the leucine derivative, regardless of the protonation state of the amino group, is the *antiperiplanar* one—carbonyl and amino/ammonium groups oriented antiperiplanar—(Figures 5a,b, and Table S2). However, after addition of the Ag^+^ ion, DFT calculations [B3LYP-D3/6-311+G(d,p) for all atoms except silver, which used B3LYP-D3/cc-pVDZ(-pp)]^36,37^ show that chelation of the metal ion with the carbonyl and amino (NH_2_) groups, through a *synperiplanar* orientation between them, is the most stable conformer. Additionally, according to the Gibbs free energy shift, the interaction with the deprotonated form in the *synperiplanar* orientation is the most stable. EFL and NCI analyses^38^ were used to analyze the noncovalent interactions. EFL studies confirmed that there are not covalent interactions, while NCI studies indicated strong noncovalent interactions between the amide carbonyl and the amino group with the Ag^+^ ion (Figures 5c,d and S40).

Similar computational DFT calculations [B3LYP-D3/6-311+G(d,p) for all atoms except silver, which used B3LYP-D3/cc-pVDZ(-pp)] were performed by exchanging the Ag^+^ ion for an AgNP consisting of Ag_7_^0^ faces.^39−41^ In this case, the *synperiplanar* orientation between the carbonyl and NH_3_^+^ is preferred for interacting with an AgNP showing a larger Gibbs free energy shift than the deprotonated (NH_2_) form (Figure 5a). ELF and NCI studies indicated a strong noncovalent interaction between the ammonium group with Ag(0) and van der Waals interactions between the amide and the hydrogen in α position with the Ag(0) (Figures 5e,f and S41).^42^

### Stimuli-Responsive Studies of Chiral AgNP(s)@Poly(*L*)-**1**

The stimuli responsiveness of the AgNP(s)@Poly(*L*)-**1** nanocomposite prepared at two different pHs (7 and 2) was studied considering the stimuli-responsive properties of the two components of the nanocomposite, the dynamic helical polymer (Poly(*L*)-**1**) and the metal nanoparticle (AgNP).

#### External Stimulus: pH

**Figure 6:**
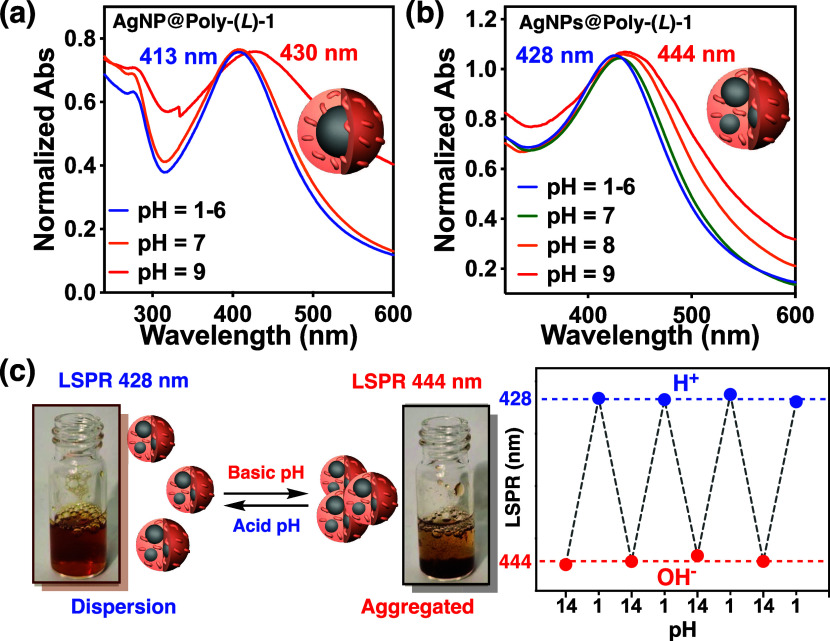
UV–vis after basification with NaOH (1M) of (a) AgNP@Poly(*L*)-**1** (previously prepared at pH = 2) and (b) AgNPs@Poly(*L*)-**1** (previously prepared at pH = 7) (c = 0.3 mg/mL of Poly(*L*)-**1**). (c) Representation of the behavior of AgNPs@Poly(*L*)-**1** with increasing pH after the addition of NaOH (aggregated) and with decreasing pH after the addition of HCl (dispersed).

Poly(*L*)-**1** adopts a preferred *P* helix (ECD_380 nm_ > 0) at neutral pH—NH_2_/NH_3_^+^ ratio > 1—while an *M* helical sense (ECD_380 nm_< 0) is induced in the polymer at acidic pH—NH_2_/NH_3_^+^ ratio < 1. Thus, the chiroptical properties of the two nanocomposites were checked by varying the pH of the water dispersions of the two AgNP@Poly(*L*)-**1** nanocomposites (0.3 mg/mL) in the ranges between 1 and 6.5. Interestingly, it was found that pH variations in the water dispersion of the AgNP(s)@Poly(*L*)-**1** nanocomposites do not affect the axial chirality adopted by Poly(*L*)-**1**. This fact is due to the stabilization of the nanocomposite through NH_3_^+^—Ag(0) interactions, which are maintained at different pHs and which keep the free amino groups, through a conformational communication mechanism, in an *antiperiplanar* orientation regardless of their protonation states. This result differs from studies carried out for a solution of Poly(*L*)-**1**, where changes in pH alter the NH_2_/NH_3_^+^ ratio and the preferred *synperiplanar* and *antiperiplanar* conformations adopted by the pendant—*synperiplanar* for NH_2_ and *antiperiplanar* for NH_3_^+^. However, although changes in the pH do not affect the chirality of the nanocomposites, their dispersion ability is reduced when the amount of NH_2_ increases (pH > 6). As a result, the plasmon band of the AgNP(s)@Poly(*L*)-**1** nanocomposites (LSPR = 428 nm of AgNPs@Poly(*L*)-**1** prepared at pH = 7, LSPR = 413 nm of AgNP@Poly(*L*)-**1** prepared at pH = 2) is red-shifted when the pH is higher than 7, *e.g*., LSPR at pH = 9 is 444 nm for AgNPs@Poly(*L*)-**1** previously prepared at pH = 7 and 430 nm for the AgNP@Poly(*L*)-**1** nanocomposite previously prepared at pH = 2. This bathochromic shift is due to the agglomeration of the nanocomposite at a basic pH (Figure 6a,b). This aggregation process is fully reversible, recovering the initial LSPR value at acidic pH and works perfectly after several pH cycles, as demonstrated by UV–vis, DLS, and SEM studies (Figures 6c, S21 and S34–S37).

#### External Stimulus: Divalent Metal Ions (M^2+^)

**Figure 7:**
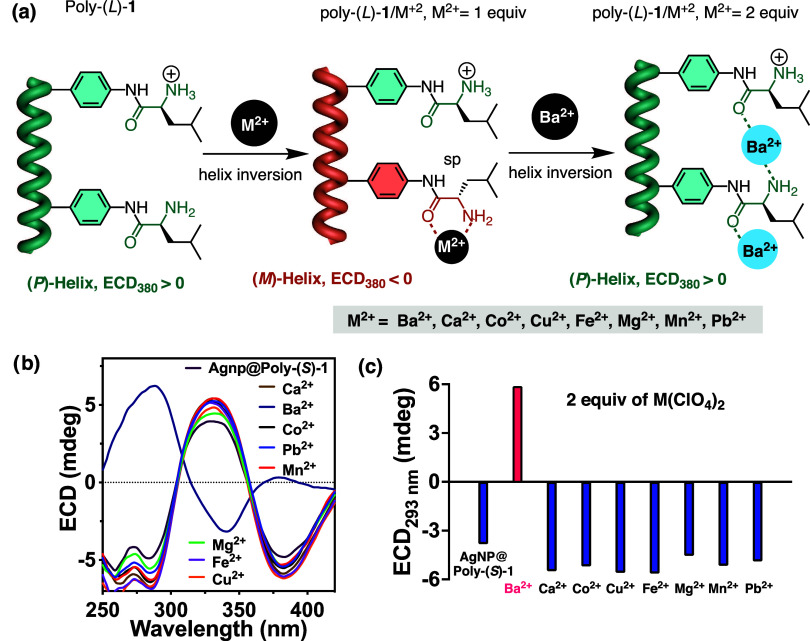
(a) Graphical illustration of the conformational changes suffered by Poly(*L*)-**1** in the presence of 1 equiv of M^2+^ and 2 equiv of Ba^2+^. (b) ECD studies of AgNP@Poly(*L*)-**1** and AgNP@Poly(*L*)-**1**/M^2+^ in 1/2 mol/mol ratios. (c) Graph bar highlighting the response of the ECD band at 293 nm of AgNP@Poly(*L*)-**1** and AgNP@Poly(*L*)-**1**/M^2+^ in 1/2 mol/mol ratios.

Poly(*L*)-**1** can interact with Ag^+^ as previously demonstrated. Thus, we decided to explore its ability to interact with other metal ions, such as divalent metal ions (M^2+^). Hence, the chiroptical properties of an aqueous solution of Poly(*L*)-**1** were explored in the presence of different divalent metal ions which were delivered to the solution as perchlorate salts [M(ClO_4_)_2_; M^2+^ = Mg^2+^, Ca^2+^, Mn^2+^, Fe^2+^, Co^2+^, Cu^2+^, Ba^2+^, and Pb^2+^]. Interestingly, it was observed that in all cases, when the metal ion is added in a 1/1 mol/mol Poly(*L*)-**1/**M(ClO_4_)_2_ ratio, a helix inversion occurs due to a chelation between the carbonyl, the amino, and the divalent metal ion that favors a *synperiplanar* conformation in the leucine pendant group (Figure 7a). Interestingly, and only in the case of Poly(*L*)-**1/**Ba^2+^, when the amount of metal ion added is 2 equiv, a second helix inversion is observed (Figure 7a and S18). This fact indicates that in the case of the other metal ions or when the Poly(*L*)-**1/**Ba^2+^ complex is in a 1/1 mol/mol ratio, the chelated form is the most favored, which orients the pendant group *synperiplanar*. On the other hand, when 2 equiv of Ba^2+^ are added to a solution of poly(*L*)-**1**—Poly(*L*)-**1/**Ba^2+^ complex in a 1/2 mol/mol ratio—an evolution toward a different complex occurs. In this special case, the adoption of an *antiperiplanar* conformation in the pendant is stabilized by the coordination of one of the Ba^2+^ ions to the carbonyl, while the other ion is coordinated to the amino group. As a result, helix inversion from *M* to *P* occurs in PPA (Figure 7a).

Analogous studies were carried out for the two nanocomposites prepared at pH = 2 and pH = 7.

Thus, different perchlorates [M(ClO_4_)_2_; M^2+^ = Mg^2+^, Ca^2+^, Mn^2+^, Fe^2+^, Co^2+^, Cu^2+^, Ba^2+^, and Pb^2+^] were added to dispersions of these nanocomposites in water in 1/1 and 1/2 mol/mol AgNP(s)@Poly(*L*)-**1/**M(ClO_4_)_2_ ratios. In these cases, Poly(*L*)-**1** adopts an *M* helix in the nanocomposites, a consequence of the interactions between carbonyl and ammonium groups with AgNPs. Thus, when 1 equiv of M(ClO_4_)_2_ is used, no effects are observed in the chirality of the nanocomposite. Both AgNPs and M^2+^ ions induce the same helical sense in the polymer used to coat the MNPs. However, when two equiv of the metal salt is added, a helical inversion is selectively observed in the case of AgNP(s)@Poly(*L*)-**1/**Ba(ClO4)_2_, similar to the effect observed in the case of Poly(*L*)-**1**. Analogous results were found for both nanocomposites prepared at pH = 2 and pH = 7, indicating that these nanomaterials can be employed to selectively sense Ba^2+^ in aqueous media.

#### External Stimulus: Oxidizing Metal Ions

**Figure 8:**
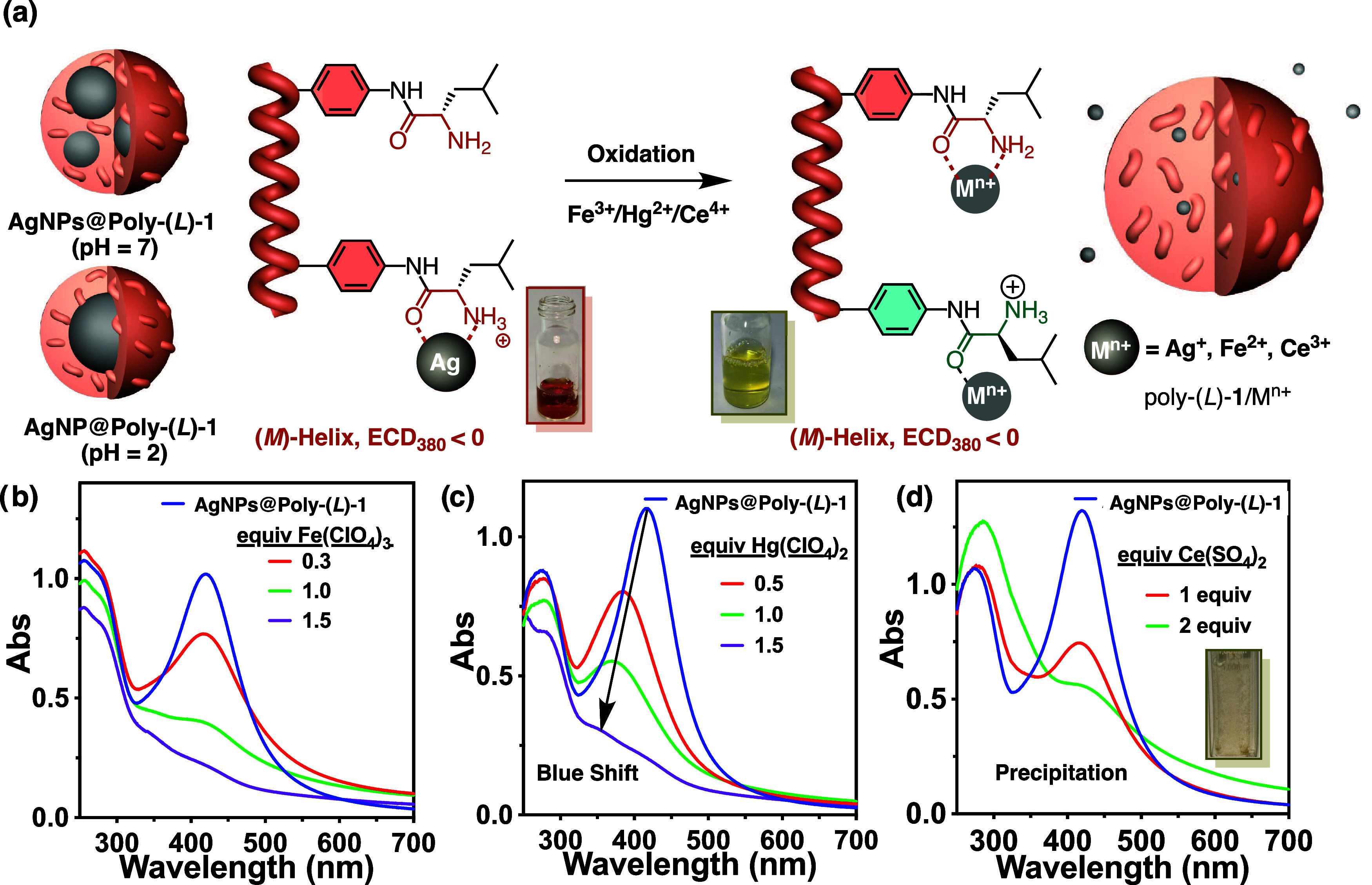
(a) Graphical illustration of the AgNP(s)@Poly(*L*)-**1** nanocomposites response toward oxidizing metal ions. UV–vis titration studies of the AgNPs@Poly(*L*)-**1** nanocomposite with (b) Fe(ClO4)_3_, (c) Hg(ClO4)_2_ and (d) Ce(SO_4_)_2_.

The optical properties of the two nanocomposites can be altered by the presence of oxidizing metal ions such as Fe^3+^, Hg^2+^, and Ce^4+^ in different ways (Figure 8a).^43−46^ Thus, while the presence of Fe^3+^ and Ce^4+^ ions in an aqueous dispersion of AgNP(s)@Poly(*L*)-**1** promotes a strong depletion of the LSPR band due to the oxidation of the AgNPs, the presence of Hg^2+^ causes a blue shift accompanied by a depletion of the LSPR band due to the formation of a Hg^0^ layer on the surface of the AgNPs once they are oxidized to Ag^+^. Thus, by observing the LSPR bands of the AgNP(s)@Poly(*L*)-**1** nanocomposites, it is possible to distinguish Hg^2+^ from the other ions (Figure 8b–d).^45^ Fortunately, Poly(L)-**1**/Ce^3+^ has a greater tendency to aggregate when compared to Poly(L)-**1**/Fe^2+^, a result that allow us to discern between these two metals and therefore between the three oxidizing ions Fe^3+^, Hg^2+^, and Ce^4+^.

In all cases, the macroscopic *M*-chirality observed in the AgNP(s)@Poly(*L*)-**1** nanocomposite is also found in the Poly(*L*)-**1**/M^n+^ complexes due to the stabilization of the *synperiplanar* conformation in the pendant by chelation between the metal ion (reduced oxidizing metal and oxidized silver) and the carbonyl and amine in the Poly-**1** pendant of the nanocomposite (Figure 8a).

## Conclusions

In this work, water-soluble and stimuli-responsive nanocomposites were prepared by combining AgNPs and a dynamic helical poly(phenylacetylene) (PPA). The designed PPA contains, as a pendant, the anilide of the amino acid alanine [Poly(*L*)-**1**], which has its N-terminus unprotected. Thus, the presence of amino groups provides the nanocomposite with the ability to disperse in water and strong Ag(0)—NH_3_^+^ interactions, necessary to stabilize the AgNPs in aqueous medium. To prepare the nanocomposite, a Poly(*L*)-**1**/Ag^+^ complex is first formed which is subsequently reduced to the desired AgNPs@Poly(*L*)-**1** nanocomposite. Interestingly, depending on the pH used to prepare the nanocomposite, different morphologies are obtained. Thus, if the nanocomposite is prepared at neutral pH (i.e., 7), then the nanocomposite consists of a ca. 250 nm nanosphere containing several 30 nm AgNPs inside. On the contrary, if the nanocomposite is prepared at acidic pH (i.e., 2), nanospheres with size of ca. 100 nm are formed containing a single 30 nm AgNP in their interior. The two nanocomposite morphologies show stimuli-responsive properties similar to those of pH and metal ions. Thus, variations in the pH lead to controlled aggregation/dispersion of the nanocomposite, resulting in variations of the LSPR band of AgNPs. This process is fully reversible, and the system works perfectly after several pH cycles. Furthermore, it was found that the AgNPs@Poly(*L*)-**1** nanocomposite can detect and identify Ba^2+^ from a series of eight different divalent metal ions [M^2+^ = Mg^2+^, Ca^2+^, Mn^2+^, Fe^2+^, Co^2+^, Cu^2+^, Ba^2+^, and Pb^2+^] delivered as perchlorate salts. Finally, we also found that the AgNPs@Poly(*L*)-**1** nanocomposite can selectively distinguish between three different oxidizing metal ions such as Fe^3+^, Hg^2+^, and Ce^4+^. These metal ions oxidize AgNPs to Ag(I) within the nanocomposite but with different responses. Thus, both Fe^3+^ and Ce^4+^ cause a strong depletion of the LSPR bands of AgNPs, but in the case of Ce^4+^, it is also accompanied by precipitation of the final product. On the other hand, the presence of Hg^2+^ ions produces a depletion of the LSPR band that is accompanied by a blue shift. In this way, water-soluble chiral AgNP(s)@PPA nanocomposites have been prepared that combine the optical and chiroptical properties of both components. The stability of these systems, in combination with the stimuli-responsive properties of the nanocomposites, allows the preparation of aqueous stimuli-responsive materials with potential applications in sensing, chirality, chemical biology, or medicinal chemistry, among others.

## Materials and Methods

Commercially available chemicals were used as delivered. Solvents were purchased as reagent grade and distilled if necessary. Anhydrous solvents were either purchased as ultradry solvent from Acros Organics or received from a solvent purification system. For the coupling and polymerization reactions, dry THF was obtained from a MBRAUN SPS 800 solvent purification system. The reactions were performed in inert atmospheres.

Column chromatography was performed with silica gel with 230–400 mesh (Merck).

Thin-layer chromatography of silica 60F254 Merck was visualized in UV at a wavelength of 254 nm and by heat with a Hanessian.

The IR spectra were obtained using the FT-IR PerkinElmer Spectrum Two equipped with the UATR add-on.

NMR experiments were measured in a Varian 300 operating at 300 MHz for proton NMR, and 75 MHz for carbon. TMS signal (δ = 0 ppm) was used as internal reference for 1H NMR experiments; CDCl3 signal (δ = 77.2 ppm) was used as a standard for 13C experiments.

The UV/vis absorption spectra were acquired in the Jasco V-730 spectrophotometer in the range of wavelengths from 240 to 500 nm. All samples were prepared at a fixed concentration of 0.3 mg of polymer/mL of solvent inside quartz glass cuvettes with a light path of 1 mm.

The ECD spectra were acquired in the Jasco 720 spectrophotometer in the range of wavelengths from 240 to 500 nm. All samples were prepared at a fixed concentration of 0.3 mg polymer/mL solvent inside quartz glass cuvettes with a light path of 1 mm. pH studies were carried out with a concentration of 0.03 mg of polymer/mL inside quartz glass cuvettes with a light path of 10 mm.

DLS measurements were performed on a Nano-ZS 90 (Malvern) equipped with a He–Ne laser (*l* = 633 nm) under a scattering angle of 173°. The samples were maintained at the designed temperature for 5 min before testing.

SEM samples were performed on a LEO-435VP electron microscope. A drop of sample was settled on a silicon wafer chip and allowed to dry at rt for 12 h.

TEM measurements were performed on a Phillips CM-12 electron microscope. A drop of sample was settled on a silicon wafer chip and allowed to dry at rt for 12 h.

Irradiation experiments were done in an Asahi Spectra Xenon light, model MAX-303. The light was filtered using a short pass filter/Vis 550 nm 25 dia.

Gaussian curve fitting was performed with Solver from excel for the electron microscopy measured sizes with the following equation:
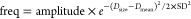


## Supporting Information Available

Materials and methods; monomers and polymers; synthetic procedure of intermediate derivated from 4-ethynylaniline; deprotection protocol and synthesis of (L)-1; polymer synthesis, CD and UV–vis of monomer (L)-1 and addition of AgClO_4_ followed by NaBH_4_; degradation of Poly(L)-1 after the interaction with NaBH_4_; screening of poly(L)-1 at pH = 7 with AgClO_4_; ECD and UV–vis studies of Poly(L/D)-1/Ag+ complexes and AgNP@Poly(L/D)-1 or AgNPs@Poly(L/D)-1 nanocomposites; synthesis of AgNP@Poly(L)-1 with more equivalents of AgClO_4_; stability studies of AgNPs@Poly(L)-1 at different temperatures and time; ECD and UV–vis studies of the oxidation of AgNP@Poly(L)-1 with Fe(ClO4)_3_, Hg(ClO4)_2_, and Ce(SO4)_2_; ECD, UV–vis, and DLS studies of the oxidation of AgNPs@Poly(L)-1 with Fe(ClO4)_3_, Hg(ClO4)_2_, and Ce(SO4)_2_; ECD and UV–vis studies of AgNPs@Poly(L)-1 in the presence of ultrapure FeCl_2_ and not ultrapure Fe(ClO4)_2_; ECD and UV–vis studies of AgNPs@Poly(L)-1 in the presence of salts; ECD study of Poly(L)-1 interaction with Ba(ClO4)_2_; chiroptical study of AgNPs@Poly(L)-1 and of AgNP@Poly(L)-1 nanocomposites in the presence of divalent perchlorate salts; UV–vis of the nanocomposites after basification; UV–vis and DLS of the nanocomposite following cycles of basification and acidification; Poly(L)-1 at different pH; scanning electron microscopy images of Poly(L)-1/AgClO4 pH = 7; scanning electron microscopy images of AgNP@Poly(L)-1 synthesized at pH = 2; transmission electron microscopy images of AgNP@ Poly(L)-1 synthesized at pH = 2; scanning electron microscopy images of AgNPs@ Poly(L)-1 synthesized at pH = 7; transmission electron microscopy images of AgNP@ Poly(L)-1 synthesized at pH = 7; scanning electron microscopy images of AgNPs@ Poly(L)-1 synthesized at pH = 7 before and after precipitation with NaOH 1M and recovery with HCl 1M; scanning electron microscopy images of AgNP@ Poly(L)-1 synthesized at pH = 2 before and after precipitation with NaOH 1M and recovery with HCl 1M; theoretical calculations; references (PDF)

## References

[ref1] Jaque D.; Martínez Maestro L.; del Rosal B.; Haro-González P.; Benayas A.; Plaza J. L.; Martín Rodríguez E.; García Solé J. Nanoparticles for photothermal therapies. Nanoscale 2014, 6, 9494–9530. 10.1039/C4NR00708E. 25030381

[ref2] Kirubaharan C.; Kalpana D.; Lee Y.; Kim A.; Yoo D.; Nahm K.; Kumar G. Green Synthesis of Silver Nanoparticles: A Review. Ind. Eng. Chem. Res. 2012, 51, 7441–7446. 10.1021/ie3003232.

[ref3] Mitsudome T.; Noujima A.; Mikami Y.; Mizugaki T.; Jitsukawa K.; Kaneda K. Supported Gold and Silver Nanoparticles for Catalytic Deoxygenation of Epoxides into Alkenes. Angew. Chem., Int. Ed. 2010, 49, 5545–5548. 10.1002/anie.201001055. 20468020

[ref4] Grzelczak M.; Vermant J.; Furst E.; Liz-Marzán L. M. Directed Self-Assembly of Nanoparticles. ACS Nano 2010, 4, 3591–3605. 10.1021/nn100869j. 20568710

[ref5] Choueiri R. M.; Klinkova A.; Thérien-Aubin H.; Rubinstein M.; Kumacheva E. Structural Transitions in Nanoparticle Assemblies Governed by Competing Nanoscale Forces. J. Am. Chem. Soc. 2013, 135, 10262–10265. 10.1021/ja404341r. 23806016 PMC3755595

[ref6] Grzelczak M.; Liz-Marzán L. M.; Klajn R. Stimuli-responsive self-assembly of nanoparticles. Chem. Soc. Rev. 2019, 48, 1342–1361. 10.1039/C8CS00787J. 30688963

[ref7] Zhang J.; J Santos P.; A Gabrys P.; Lee S.; Liu C.; J Macfarlane R. Self-Assembling Nanocomposite Tectons. J. Am. Chem. Soc. 2016, 138, 16228–16231. 10.1021/jacs.6b11052. 27935680

[ref8] Li C.-C.; Chang S.-J.; Su F.-J.; Lin S.; Chou Y.-C. Effects of capping agents on the dispersion of silver nanoparticles. Colloids Surf., A 2013, 419, 209–215. 10.1016/j.colsurfa.2012.11.077.

[ref9] Pigliacelli C.; Sánchez-Fernández R.; García M. D.; Peinador C.; Pazos E. Self-assembled peptide–inorganic nanoparticle superstructures: from component design to application. Chem. Commun. 2020, 56, 8000–8014. 10.1039/d0cc02914a. 32495761

[ref10] Lee H.-E.; Ahn H.-Y.; Mun J.; Lee Y. Y.; Kim M.; Cho N. H.; Chang K.; Kim S.; Rho W. J.; Nam K. T. Amino-acid- and peptide-directed synthesis of chiral plasmonic gold nanoparticles. Nature 2018, 556, 360–365. 10.1038/s41586-018-0034-1. 29670265

[ref11] González-Rubio G.; Mosquera J.; Kumar V.; Pedrazo-Tardajos A.; Llombart P.; Solís D.; Lobato I.; Noya E.; Guerrero-Martínez A.; Taboada J.; Obelleiro F.; Macdowell L.; Bals S.; Liz-Marzán L. M. Micelle-directed chiral seeded growth on anisotropic gold nanocrystals. Science 2020, 368, 1472–1477. 10.1126/science.aba0980. 32587018

[ref12] Lu J.; Xue Y.; Bernardino K.; Zhang N.; Gomes W.; Ramesar N.; Liu S.; Hu Z.; Sun T.; de Moura A. F.; Kotov N.; Liu K. Enhanced optical asymmetry in supramolecular chiroplasmonic assemblies with long-range order. Science 2021, 371, 1368–1374. 10.1126/science.abd8576. 33632891

[ref13] Zhang N.-N.; Sun H.-R.; Liu S.; Xing Y.-C.; Lu J.; Peng F.; Han C.-L.; Wei Z.; Sun T.; Yang B.; Liu K. Gold Nanoparticle Enantiomers and Their Chiral-Morphology Dependence of Cellular Uptake. CCS Chem. 2022, 4, 660–670. 10.31635/ccschem.021.202000637.

[ref14] Lee J.-S.; Lytton-Jean A.; Hurst S.; Mirkin C. Silver nanoparticle - Oligonucleotide conjugates based on DNA with triple cyclic disulfide moieties. Nano Lett. 2007, 7, 2112–2115. 10.1021/nl071108g. 17571909 PMC3200546

[ref15] Pazos E.; Sleep E.; Pérez C.; Lee S.; Tantakitti F.; Stupp S. Nucleation and Growth of Ordered Arrays of Silver Nanoparticles on Peptide Nanofibers: Hybrid Nanostructures with Antimicrobial Properties. J. Am. Chem. Soc. 2016, 138, 5507–5510. 10.1021/jacs.6b01570. 27103596 PMC4859321

[ref16] Porrelli D.; Mardirossian M.; Musciacchio L.; Pacor M.; Berton F.; Crosera M.; Turco G. Antibacterial Electrospun Polycaprolactone Membranes Coated with Polysaccharides and Silver Nanoparticles for Guided Bone and Tissue Regeneration. ACS Appl. Mater. Interfaces 2021, 13, 17255–17267. 10.1021/acsami.1c01016. 33822574

[ref17] Núñez-Martínez M.; Arias S.; Quiñoá E.; Riguera R.; Freire F. Dynamic Chiral PPA–AgNP Nanocomposites: Aligned Silver Nanoparticles Decorating Helical Polymers. Chem. Mater. 2021, 33, 4805–4812. 10.1021/acs.chemmater.1c00805.

[ref18] Núñez-Martínez M.; Quiñoá E.; Freire F. Chiroptical and colorimetric switches based on helical polymer-metal nanocomposites prepared *via* redox metal translocation of helical polymer metal complexes. Nanoscale 2022, 14, 13066–13072. 10.1039/D2NR03807B. 36069960

[ref19] Núñez-Martínez M.; Arias S.; Bergueiro J.; Quiñoá E.; Riguera R.; Freire F. The Role of Polymer–AuNP Interaction in the Stimuli-Response Properties of PPA–AuNP Nanocomposites. Macromol. Rapid Commun. 2022, 43, 2100616 10.1002/marc.202100616. 34761481

[ref20] Núñez-Martínez M.; Quiñoá E.; Freire F. Stereocomplex Nanocomposite Switch Based on Dynamic Helical Polymer-Gold and Silver Nanoparticle Hybrid Materials. Chem. Mater. 2023, 35, 4865–4872. 10.1021/acs.chemmater.3c00912.

[ref21] Yashima E.; Maeda K.; Lida H.; Furusho Y.; Nagai K. Helical Polymers: Synthesis, Structures, and Functions. Chem. Rev. 2009, 109, 6102–6211. 10.1021/cr900162q. 19905011

[ref22] Yashima E.; Ousaka N.; Taura D.; Shimomura K.; Ikai T.; Maeda K. Supramolecular Helical Systems: Helical Assemblies of Small Molecules, Foldamers, and Polymers with Chiral Amplification and Their Functions. Chem. Rev. 2016, 116, 13752–13990. 10.1021/acs.chemrev.6b00354. 27754649

[ref23] Lago-Silva M.; Fernández-Míguez M.; Rodríguez R.; Quiñoá E.; Freire F. Stimuli-responsive Synthetic Helical Polymers. Chem. Soc. Rev. 2024, 53, 793–852. 10.1039/D3CS00952A. 38105704

[ref24] Rey-Tarrío F.; Rodríguez R.; Quiñoá E.; Riguera R.; Freire F. Photochemical Electrocyclization of Poly(phenylacetylene)s: Unwinding Helices to Elucidate their 3D Structure in Solution. Angew. Chem., Int. Ed. 2021, 60, 8095–8103. 10.1002/anie.202014780. 33332770

[ref25] Rodríguez R.; Suárez-Picado E.; Quiñoá E.; Riguera R.; Freire F. A Stimuli-Responsive Macromolecular Gear: Interlocking Dynamic Helical Polymers with Foldamers. Angew. Chem., Int. Ed. 2020, 59, 8616–8622. 10.1002/anie.201915488. 32145047

[ref26] Leiras S.; Suárez-Picado E.; Quiñoá E.; Riguera R.; Freire F. Tuning the helical sense and elongation of polymers through the combined action of the two components of tetraalkylammonium-anion salts. Giant 2021, 7, 100068 10.1016/j.giant.2021.100068.

[ref27] Lago M.; Cid M.; Quiñoá E.; Freire F. Dynamic Axial-to-Helical Communication Mechanism in Poly[(allenylethynylenephenylene)acetylene]s under External Stimuli. Angew. Chem., Int. Ed. 2023, 62, e2023003329 10.1002/anie.202303329. 37213135

[ref28] Rey-Tarrío F.; Rodríguez R.; Quiñoá E.; Freire F. Screw sense excess and reversals of helical polymers in solution. Nat. Commun. 2023, 14, 1742 10.1038/s41467-023-37405-z. 36990975 PMC10060220

[ref29] Rey-Tarrío F.; Guisán-Ceinós S.; Cuerva J.; Miguel D.; Ribagorda M.; Quiñoá E.; Freire F. Photostability and Dynamic Helical Behavior in Chiral Poly(phenylacetylene)s with a Preferred Screw-Sense. Angew. Chem., Int. Ed. 2022, 61, e202207623 10.1002/anie.202207623. PMC9543806 35731840

[ref30] Suárez-Picado E.; Quiñoá E.; Riguera R.; Freire F. Chiral Overpass Induction in Dynamic Helical Polymers Bearing Pendant Groups with Two Chiral Centers. Angew. Chem., Int. Ed. 2020, 59, 4537–4543. 10.1002/anie.201915213. 31880378

[ref31] Núñez-Martínez M.; Fernández-Míguez M.; Quiñoá E.; Freire F. Size Control of Chiral Nanospheres Obtained via Nanoprecipitation of Helical Poly(phenylacetylene)s in the Absence of Surfactants. Angew. Chem., Int. Ed. 2024, 63, e202403313 10.1002/anie.202403313. 38742679

[ref32] Suárez-Picado E.; Quiñoá E.; Riguera R.; Freire F. Poly(phenylacetylene) Amines: A General Route to Water-Soluble Helical Polyamines. Chem. Mater. 2018, 30, 6908–6914. 10.1021/acs.chemmater.8b03238.

[ref33] Szakács Z.; Kraszni M.; Noszal B. Determination of microscopic acid–base parameters from NMR–pH titrations. Anal. Bioanal. Chem. 2004, 378, 1428–1448. 10.1007/s00216-003-2390-3. 15214406

[ref34] Weisell J.; Hyvönen M. T.; Vepsäläinen J.; Alhonen L.; Keinänen T. A.; Khomutov A. R.; Soininen P. Novel isosteric charge-deficient spermine analogue—1,12-diamino-3,6,9-triazadodecane: synthesis, p*K*_a_ measurement and biological activity. Amino Acids 2010, 38, 501–507. 10.1007/s00726-009-0409-6. 19953281

[ref35] Noszál B.; Guo W.; Rabenstein D. L. Characterization of the macroscopic and microscopic acid-base chemistry of the native disulfide and reduced dithiol forms of oxytocin, arginine-vasopressin, and related peptides. J. Org. Chem. 1992, 57, 2327–2334. 10.1021/jo00034a026.

[ref36] Schneebeli S. T.; Bochevarov A. D.; Friesner R. A. Parameterization of a B3LYP specific correction for non-covalent interactions and basis set superposition error on a gigantic dataset of CCSD(T) quality non-covalent interaction energies. J. Chem. Theory Comput. 2011, 7, 658–668. 10.1021/ct100651f. 22058661 PMC3206731

[ref37] Khavani S. M.; Mehranfar A.; Izadyar M. A theoretical approach on the ability of functionalized gold nanoparticles for detection of Cd^2+^. Sci. Rep. 2021, 11, 23422 10.1038/s41598-021-02933-5. 34873260 PMC8648727

[ref38] Johnson E. R.; Keinan S.; Mori-Sánchez P.; Contreras-García J.; Cohen A.; Yang W. Revealing Noncovalent Interactions. J. Am. Chem. Soc. 2010, 132, 6498–6506. 10.1021/ja100936w. 20394428 PMC2864795

[ref39] Nhat P. V.; Si N. T.; Nguyen M. T. Comment on “Theoretical Investigations on Geometrical and Electronic Structures of Silver Clusters". J. Comput. Chem. 2019, 40, 1990–1993. 10.1002/jcc.25849. 31063638

[ref40] Rodríguez-Kessler P. L.; Rodríguez-Domínguez A. R.; Carey D. M.; Muñoz-Castro A. Structural characterization, reactivity, and vibrational properties of silver clusters: a new global minimum for Ag_16_. Phys. Chem. Chem. Phys. 2020, 22, 27255–27262. 10.1039/D0CP04018E. 33227109

[ref41] Nhat P. V.; Si N. T.; Nguyen M. T. Elucidation of the molecular and electronic structures of some magic silver clusters Ag_*n*_(*n* = 8, 18, 20). J. Mol. Model. 2018, 24, 209 10.1007/s00894-018-3730-8. 30022315

[ref42] Boto R. A.; Contreras-García J.; Calatayud M. The role of dispersion forces in metal-supported self-assembled monolayers. Comput. Theor. Chem. 2015, 1053, 322–327. 10.1016/j.comptc.2014.10.015.

[ref43] Balasurya S.; Syed A.; Thomas A.; Marraiki N.; Elgorban A.; Raju L.; Das A.; Khan S. Rapid colorimetric detection of mercury using silver nanoparticles in the presence of methionine. Spectrochim. Acta, Part A 2020, 228, 117712 10.1016/j.saa.2019.117712. 31753653

[ref44] Zou Y.; Pang J.; Zhang F.; Chai F. Silver Nanoparticles for Colorimetric Detection and Discrimination of Mercury Ions in Lake Water. ChemistrySelect 2021, 6, 6077–6082. 10.1002/slct.202101389.

[ref45] Schiesaro I.; Burrati L.; Meneghini C.; Fratoddi I.; Proposito P.; Lim J.; Scheu C.; Venditti I.; Iucci G.; Battocchio C. Hydrophilic Silver Nanoparticles for Hg(II) Detection in Water: Direct Evidence for Mercury–Silver Interaction. J. Phys. Chem. C 2020, 124, 25975–25983. 10.1021/acs.jpcc.0c06951.

[ref46] Maity M.; Bera K.; Pal U.; Khamary K.; N Maiti N. Sensing of Iron(III) Ion via Modulation of Redox Potential on Biliverdin Protected Silver Nanosurface. ACS Appl. Nano Mater. 2018, 1, 6099–6111. 10.1021/acsanm.8b01311.

