# Supplementary material for: Optical and Chiroptical Stimuli-Responsive Chiral AgNPs@H-Leu-Poly(phenylacetylene) Nanocomposites in Water
Source: ACS Nano. 2024 Oct 9;18(42):28822–33. doi: 10.1021/acsnano.4c08622 (PMC11503914; doi:10.1021/acsnano.4c08622)
Supplement: Supplementary file 1 — nn4c08622_si_001.pdf [file nn4c08622_si_001.pdf]

**Optical and Chiroptical Stimuli-Responsive Chiral AgNPs@H-Leu-Poly(phenylacetylene) Nanocomposites in Water**

*Manuel Fernández-Míguez, Manuel Núñez-Martínez, Esteban Suárez-Picado, Emilio Quiñoá and Félix Freire**

*Centro Singular de Investigación en Química Biolóxica e Materiais Moleculares (CiQUS) and Departamento de Química Orgánica*

*Universidade de Santiago de Compostela*

*15782 Santiago de Compostela (Spain)*

*E-mail: felix.freire@usc.es*

## Table of contents


**1. Monomers and polymers ........................................................................................ 4**

**2. Synthetic procedure of intermediate derivated from 4-ethynylaniline ............................... 5**

**3. Deprotection protocol and synthesis of (*L*)-1 .................................................................. 7**

**4. Polymer synthesis ........................................................................................................ 9**

**5. CD and UV-Vis of Monomer (*L*)-1 and addition of $AgClO_4$ followed by $NaBH_4$. ....................10**

**6. Screening of poly-(*L*)-1 at pH = 7 with $AgClO_4$. ..................................................................10**

**7. ECD and UV-Vis studies of Poly-(*L/D*)-1/$Ag^+$ Complexes and AgNP@Poly-(*L*/*D*)-1 or AgNPs@Poly-(*L*/*D*)-1 nanocomposites. ........................................................................11**

**8. Synthesis of AgNP@Poly-(*L*)-1 with more equivalents of $AgClO_4$ ......................................12**

**9. Thermal, Time and Photo Stability studies of Poly-(*L*)-1and AgNPs@Poly-(*L*)-1 ..................13**

**10. ECD and UV-Vis studies of the oxidation of AgNP@Poly-(*L*)-1 with $Fe(ClO_4)_3$, $Hg(ClO_4)_2$ and $Ce(SO_4)_2$. ..............................................................................................................14**

**11. ECD, UV-Vis and DLS studies of the oxidation of AgNPs@Poly-(*L*)-1 with $Fe(ClO_4)_3$, $Hg(ClO_4)_2$ and $Ce(SO_4)_2$. ..............................................................................................15**

**12. ECD and UV-Vis studies of AgNPs@Poly-(*L*)-1 in the presence of ultrapure $FeCl_2$ and not ultrapure $Fe(ClO_4)_2$. .............................................................................................16**

**13. ECD and UV-Vis studies of AgNPs@Poly-(*L*)-1 in the presence of salts. ............................17**

**14. ECD study of Poly-(*L*)-1 interaction with $Ba(ClO_4)_2$. .........................................................18**

**15. Chiroptical study of AgNPs@Poly-(*L*)-1 and of AgNP@Poly-(*L*)-1 nanocomposites in the presence of divalent perchlorate salts. ........................................................................18**

**16. UV-Vis of the nanocomposites after basification. ............................................................19**

**17. UV-Vis and DLS of the nanocomposite following cycles of basification and acidification. .19**

**18. Poly-(*L*)-1 at different pH. ..............................................................................................20**

**19. Scanning electron microscopy images of Poly-(*L*)-1/$AgClO_4$ pH = 7. ..................................20**

**20. Scanning electron microscopy images of AgNP@Poly-(*L*)-1 synthetized at pH = 2. ............21**

**21. Transmission electron microscopy images of AgNP@ Poly-(*L*)-1 synthetized at pH = 2. ....21**

**22. Scanning electron microscopy images of AgNPs@ Poly-(*L*)-1 synthetized at pH = 7. .........22**

**23. Transmission electron microscopy images of AgNP@ Poly-(*L*)-1 synthetized at pH = 7. ....23**

**24. Scanning electron microscopy images of AgNPs@ Poly-(*L*)-1 synthetized at pH = 7 before after precipitation with NaOH 1M and recovery with HCl 1M ...............................................24**

**25. Scanning electron microscopy images of AgNP@ Poly-(*L*)-1 synthetized at pH = 2 before after precipitation with NaOH 1M and recovery with HCl 1M ............................................25**

**26. UV-Vis and TEM studies of AgNP@Poly-(*L*)-1 nanocomposite prepared (a) at pH = 7 and measured at pH = 2 (b) prepared at pH = 2 and measured at pH = 7......................................26**

**27. Theoretical Calculations...................................................................................27**

**28. References...........................................................................................................32**

## 1. Monomers and polymers

The preparation of monomers (*L*)-**1** and polymers poly-(*L*)-**1** can be found in reference [S1]. The preparation of monomer (*D*)-**1** and polymer poly-(*D*)-**1** followed the same synthesis procedures as (*L*)-**1** and poly-(*L*)-**1**.

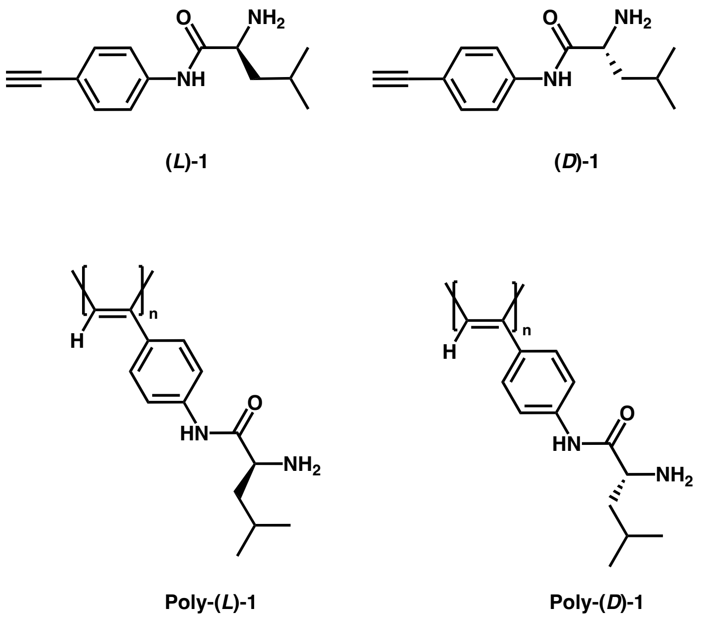


**Figure S1.** Structures of monomers and Polymers.

## 2. Synthetic procedure of intermediate derivated from 4-ethynylaniline

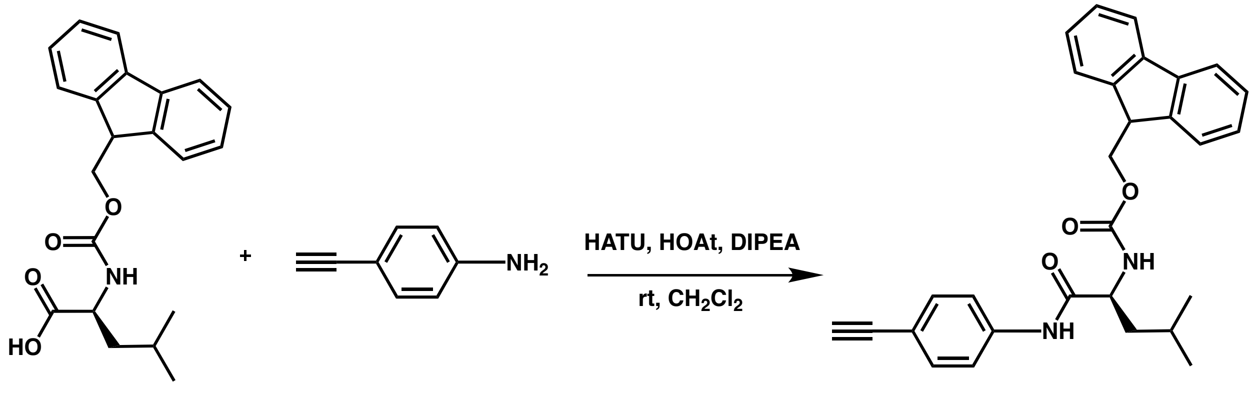


2-(7-Aza-1H-benzotriazole-1-yl)-1,1,3,3-tetramethyluronium (0.55 g, HATU, 1.2 equiv.), 1-hydroxy-7-azabenzotriazole (0,19 g, HOAt, 1.2 equiv.), **Fmoc-(*L*)-Leu** (0.5 g, 1.2 equiv.) and diisopropylethylamine (DIPEA, 0.5 mL, 1.4 equiv.) were dissolved in 20 mL of dry $CH_2Cl_2$, and the mixture was stirred for 15 min to activate the acid. Then, 4- ethynylaniline (0.14 g, 1.0 equiv.) was added and the reaction mixture was stirred overnight. Then, the organic layer was washed with HCl 1M and brine. The combined organic layers were dried over anhydride $Na_2SO_4$, filtered and the solvent was evaporated at reduced pressure. The crude product was chromatographed on silica gel (70-230 mesh) with hexane/ethyl acetate (8/2) as eluent, yields 82% of pure product (0.53 g).

$^{1}$H NMR (300 MHz, CDCl3) δ(ppm): 0.89 (m, 6H), 1.68 (m, 3H), 3.03 (s, 1H), 4.10 (m, 1H), 4.32 (m, 3H), 5.83 (m, 1H), 7.17- 7.49 (m, 9H), 7.50-7.73 (m, 3H), 8.95 (s, 1H).

$^{13}$C NMR (75 MHz, $CDCl_3$) δ (ppm): 21.9, 22.9, 24.7, 40.9, 47.0, 54.4, 67.4, 76.8, 83.4, 113.7,117.8, 119.5, 120.0, 127.1, 127.8, 130.8, 132.8, 138.2, 141.3, 143.4, 156.9, 171.1.

HRMS (ESI) m/z calcd for $C_{29}H_{28}N_2O_3$ [M+H] 453.2173, found 453.2175.

$[\alpha]_D$= -24 (c= 15 mg/mL, $CHCl_3$.

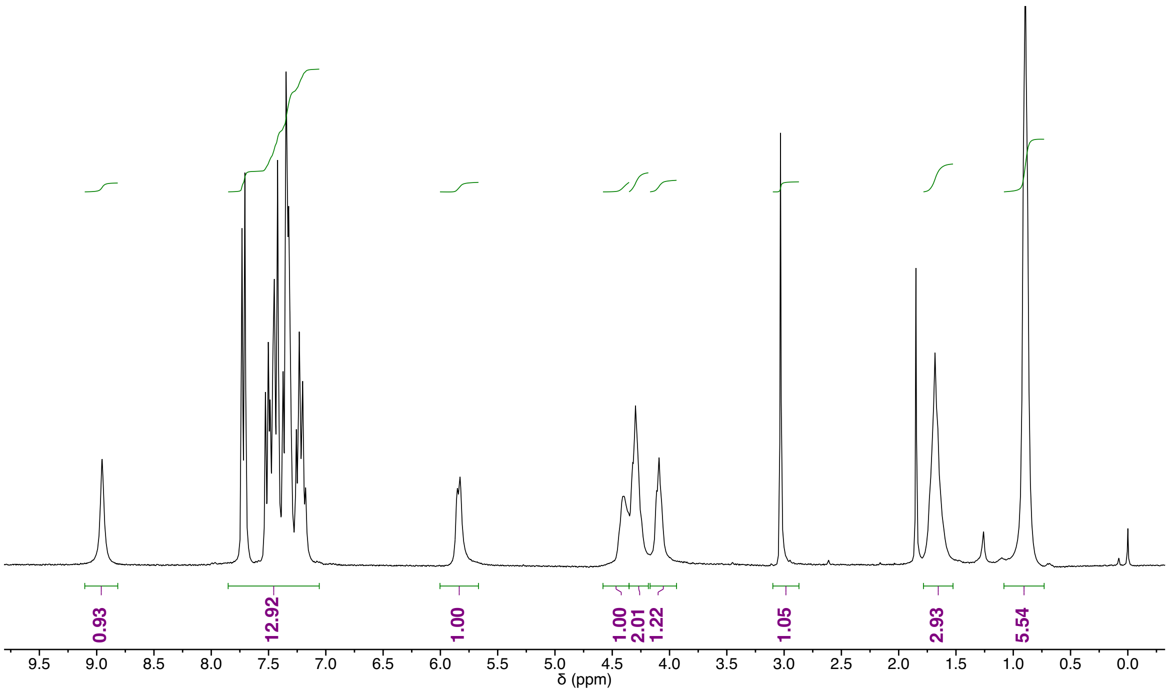


**Figure S2.** $^{1}$H-NMR spectrum of intermediate derived from 4-ethynylaniline ($CDCl_3$, 300 MHz).

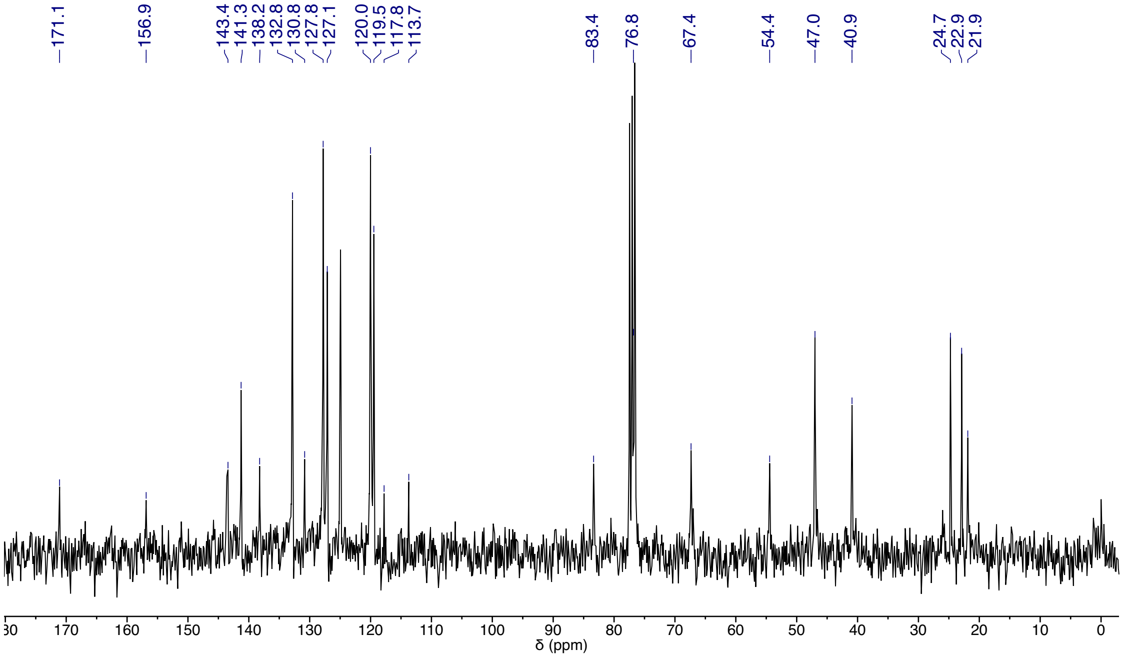


**Figure S3.** $^{13}$C-NMR spectrum of intermediate derived from 4-ethynylaniline ($CDCl_3$, 75 MHz).

### 3. Deprotection protocol and synthesis of (*L*)-1

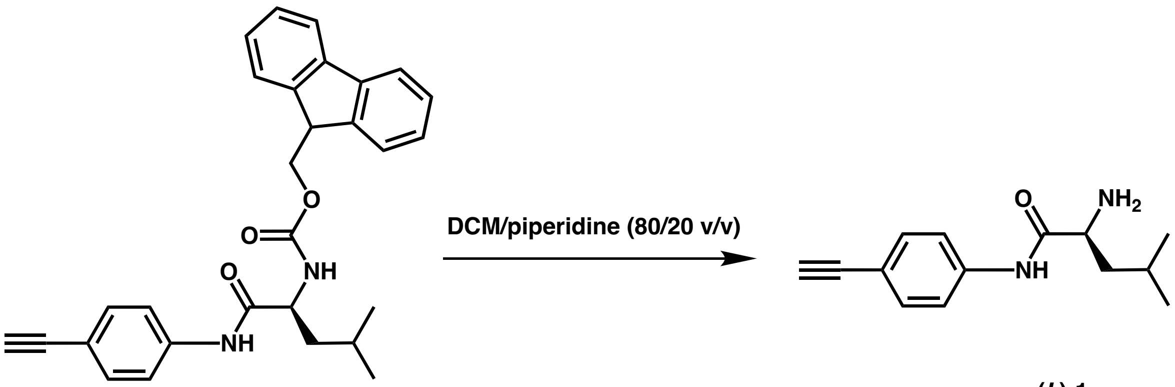


(*L*)-1

The Fmoc group of the intermediate derivated from 4-ethynylaniline was removed in a mixture of DCM/piperidine (80/20 v/v) 20%. The reaction mixture was stirred at room temperature until the reaction ended (1h). After the completion, the crude products were chromatographed on silica gel (70- 230 mesh) with hexane/ethyl acetate (1/1) as eluent, yields 72% of pure product (0.201 g).

$^1$H NMR (300 MHz, $CDCl_3$) δ(ppm): 0.96 (m, 6H), 1.44 (m, 2H), 1.78 (m, 2H), 2.56 (s, 2H), 3.03 (s, 1H), 3.60 (m, 1H), 7.43 (d, 2H), 7.54 (d, 2H), 9.69 (s, 1H).

$^{13}$C NMR (75 MHz, $CDCl_3$) δ (ppm): 21.3, 23.4, 24.9, 43.8, 53.9, 76.7, 83.5, 117.2, 118.9, 129.7, 132.8, 138.4, 173.9.

HRMS (ESI) m/z calcd for $C_{14}H_{18}N_2O$ [M+H] 231.1492, found 231.1491. $[\alpha]_D$= -43 (c= 15 mg/mL, $CHCl_3$).

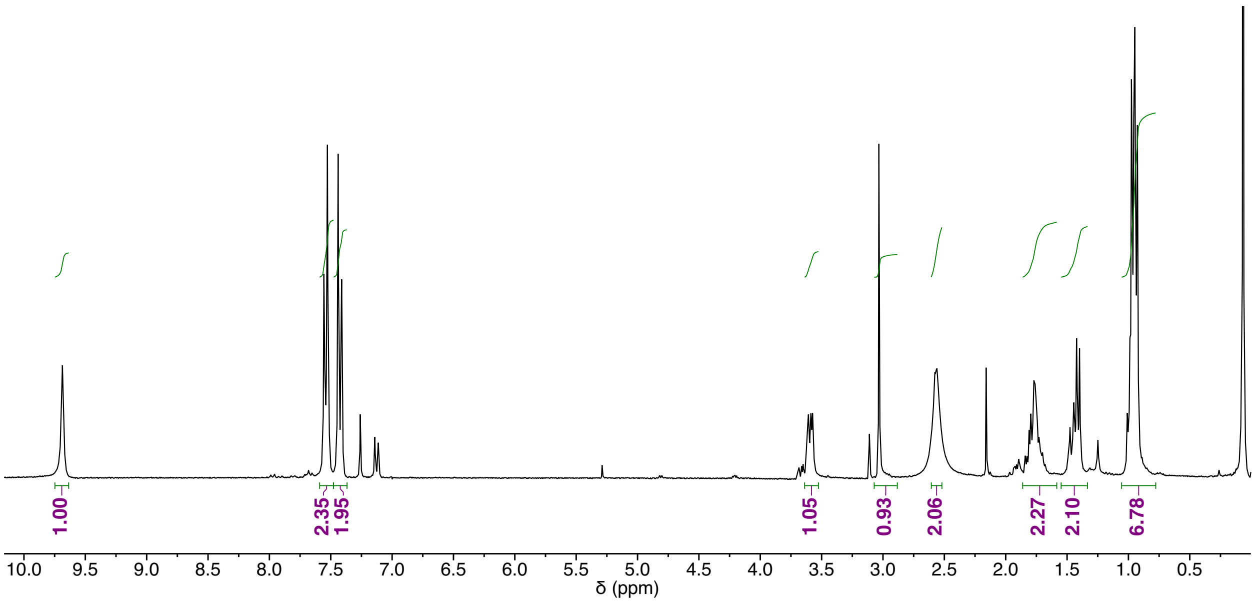


**Figure S4.** $^{1}$H-NMR spectrum of (*L*)-**1** ($CDCl_3$, 300 MHz).

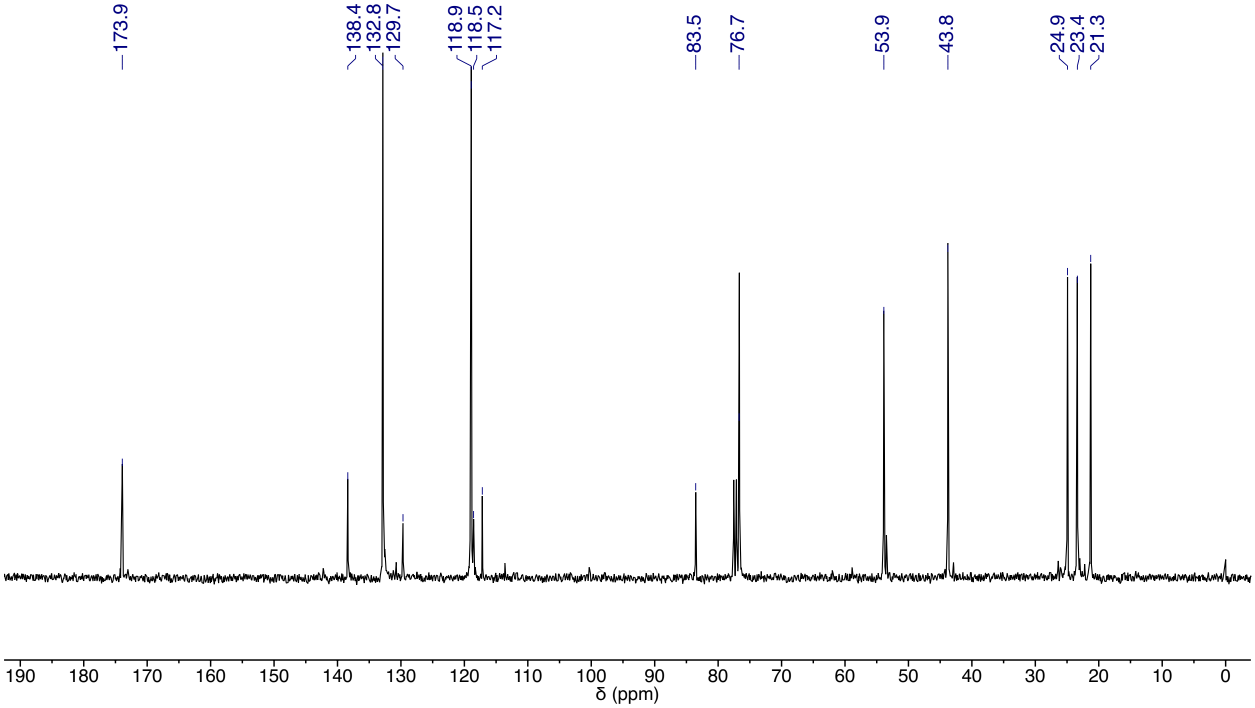


**Figure S5.** $^{13}$C-NMR spectrum of (*L*)-**1** ($CDCl_3$, 75 MHz).

## 4. Polymer synthesis

monomer (*L*)-**1** (60 mg) was added as a solid in a flask. Water (459uL) and HCl (21.5uL from 11 M, 1.0 eq.) were added in order to protonate the amino group and avoid the catalyst poisoning. Next, a solution of $[Rh(cod)_2]BF_4$ in (0,525 mg) water at r.t. The reaction mixture was stirred at r.t. for 36h. Next, the resulting polymers were diluted in water and lyophilized. After that, the polymers were dissolved in MeOH and precipitated in large amount of ethyl acetate and centrifuged twice (53.3 mg Poly-(*L*)-**1**), yields 89% of pure product.

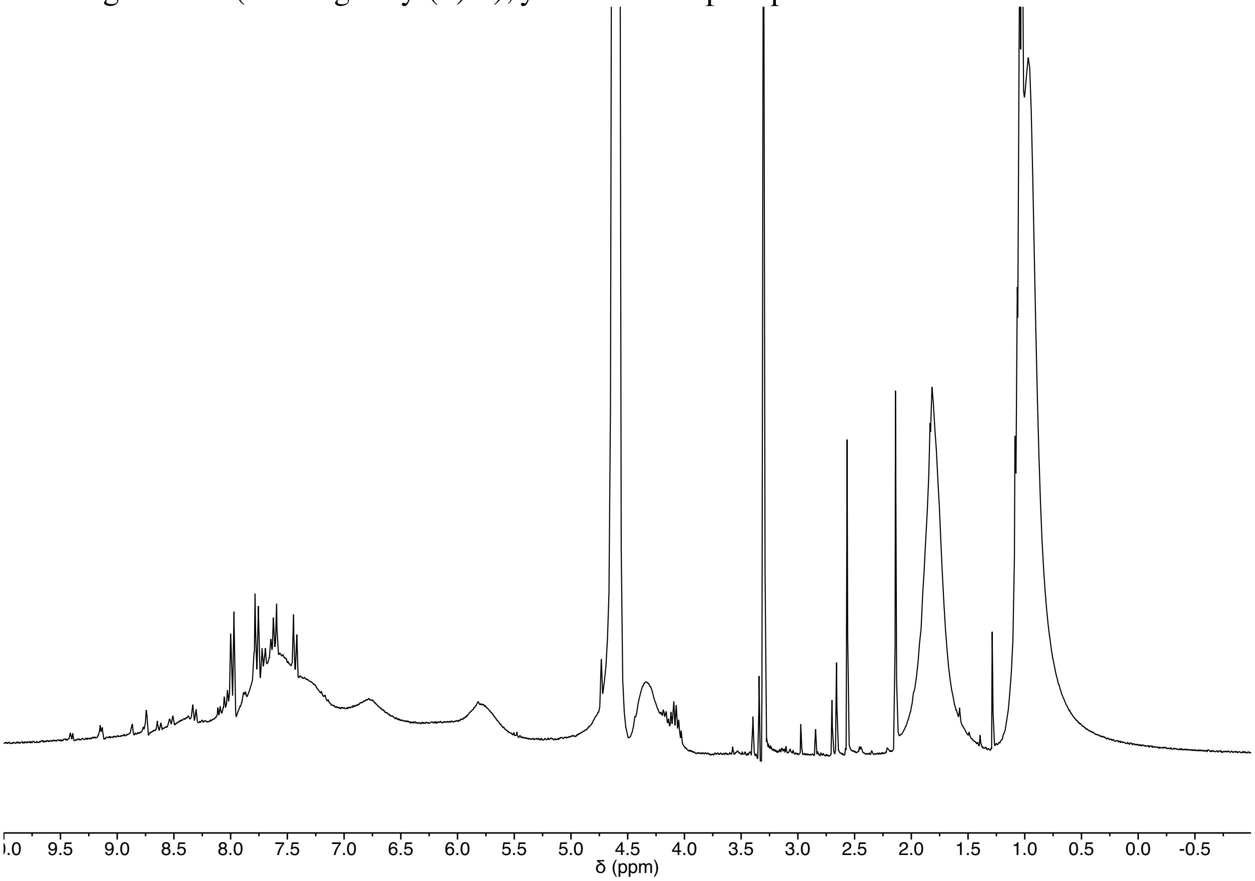


**Figure S6.** $^1$H-NMR spectrum of Poly-(*L*)-**1** (methanol-d$_4$, 300 MHz).

**Table S1.** GPC data of Poly-(*L*)-**1**

| Compound | Mn | Mw | Mp | Mz | Đ |
|---|---|---|---|---|---|
| Poly-(*L*)-**1** | 67796 | 217186 | 236216 | 330460 | 3.20 |

## 5. CD and UV-Vis of Monomer (*L*)-1 and addition of $AgClO_4$ followed by $NaBH_4$.

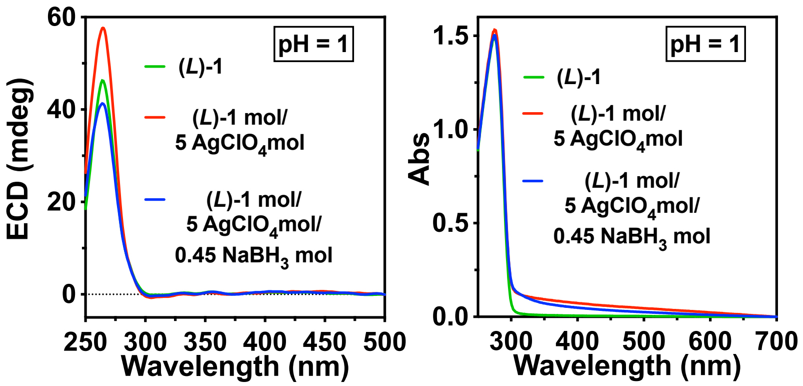


**Figure S7.** ECD and UV-Vis of (*L*)-**1** at pH = 1 in presence of 5 equivalents of $AgClO_4$ salt (10 mg $mL^{-1}$ $H_2O$ MQ) and 0.45 equivalents of $NaBH_4$ (10 mg $mL^{-1}$ $H_2O$ MQ).

## 6. Screening of poly-(*L*)-1 at pH = 7 with $AgClO_4$.

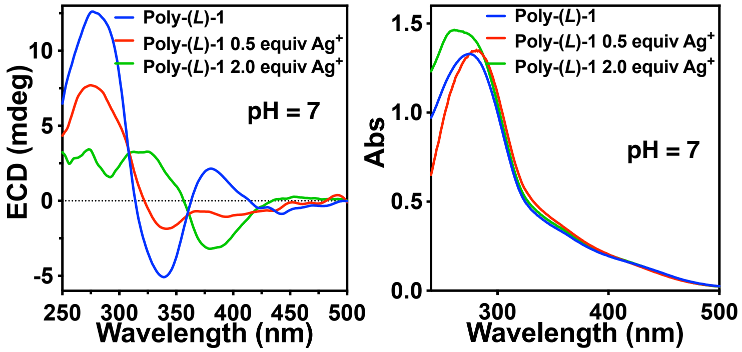


**Figure S8.** ECD and UV-Vis of poly-(*L*)-**1** (0.3 mg $mL^{-1}$ $H_2O$ MQ pH = 7) in presence of 0.5 and 2.0 equivalents of $AgClO_4$ (10 mg $mL^{-1}$ $H_2O$ MQ).

**7. ECD and UV-Vis studies of Poly-(*L*/*D*)-1/Ag$^+$ Complexes and AgNP@Poly-(*L*/*D*)-1 or AgNPs@Poly-(*L*/*D*)-1 nanocomposites.**

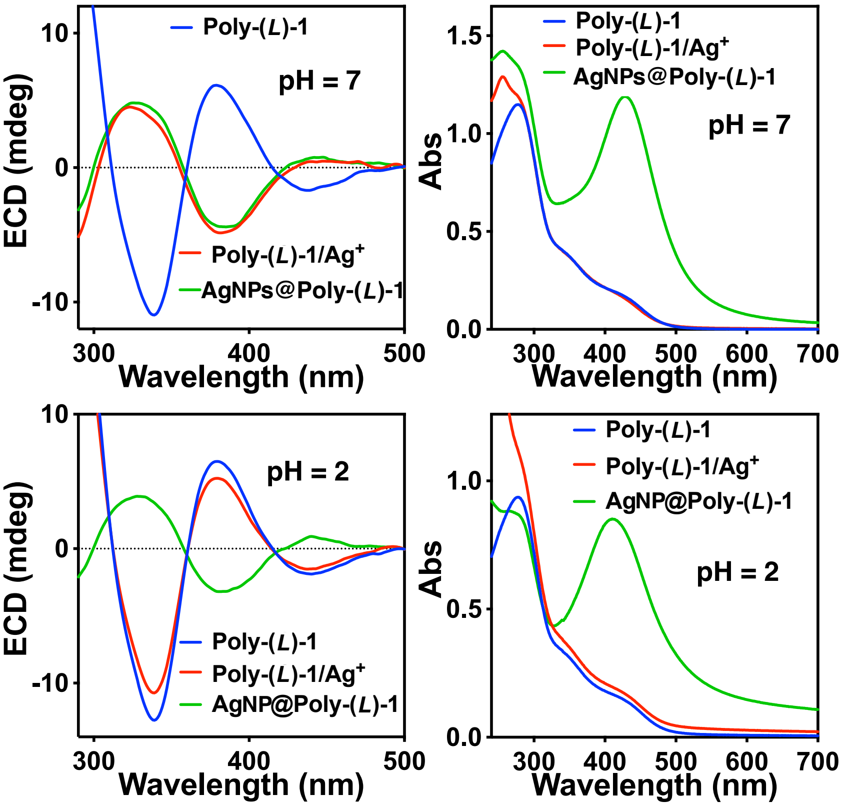


**Figure S9.** ECD and UV-Vis of poly-(*L*)-**1** (0.3 mg mL$^{-1}$ $H_2O$ MQ pH = 2 and 7) in presence of 1.5 equivalents of $AgClO_4$ (10 mg mL$^{-1}$ $H_2O$ MQ) and after the reduction with 0.45 equivalents of $NaBH_4$ (0.5 mg mL$^{-1}$ $H_2O$ MQ).

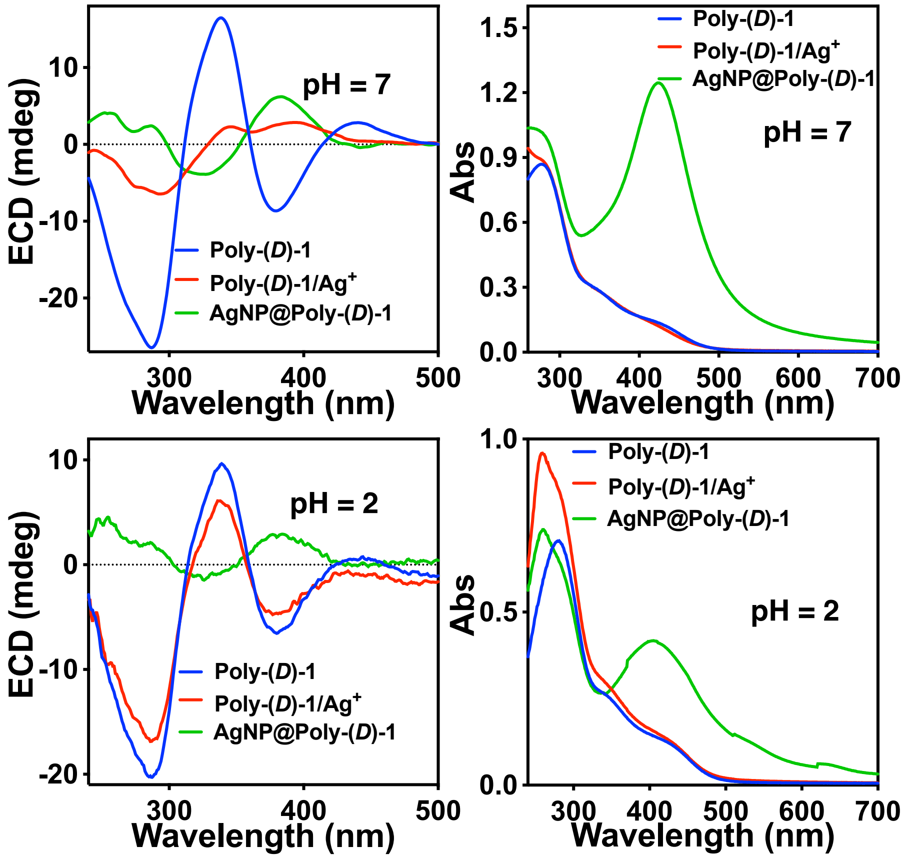


**Figure S10.** CD and UV-Vis of poly-(*D*)-**1** (0.3 mg mL$^{-1}$ $H_2O$ MQ pH = 2 and 7) in presence of 1.5 equivalents of $AgClO_4$ (10 mg mL$^{-1}$ $H_2O$ MQ) and after the reduction with 0.45 equivalents of $NaBH_4$ (0.5 mg mL$^{-1}$ $H_2O$ MQ).

## 8. Synthesis of AgNP@Poly-(*L*)-1 with more equivalents of $AgClO_4$

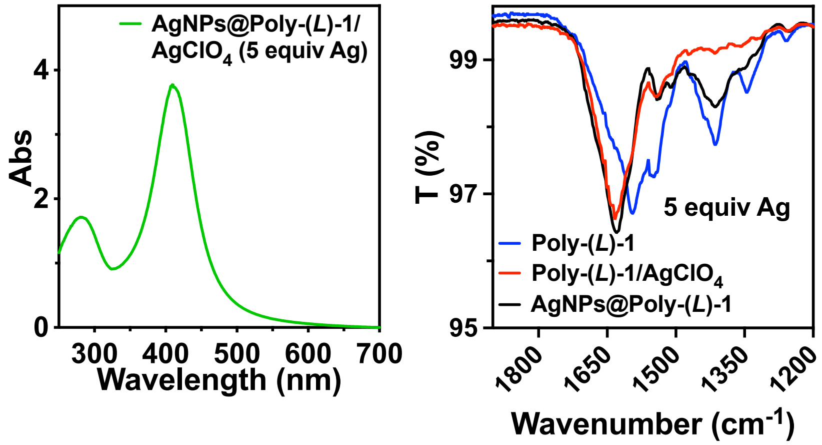


**Figure S11.** UV-Vis and FT-IR of Poly-(*L*)-**1** in presence of 5 equivalents of $AgClO_4$ salt (10 mg mL$^{-1}$ $H_2O$ MQ) and 0.45 equivalents of $NaBH_4$ (10 mg mL$^{-1}$ $H_2O$ MQ).

## 9. Thermal, Time and Photo Stability studies of Poly-(*L*)-1and AgNPs@Poly-(*L*)-1

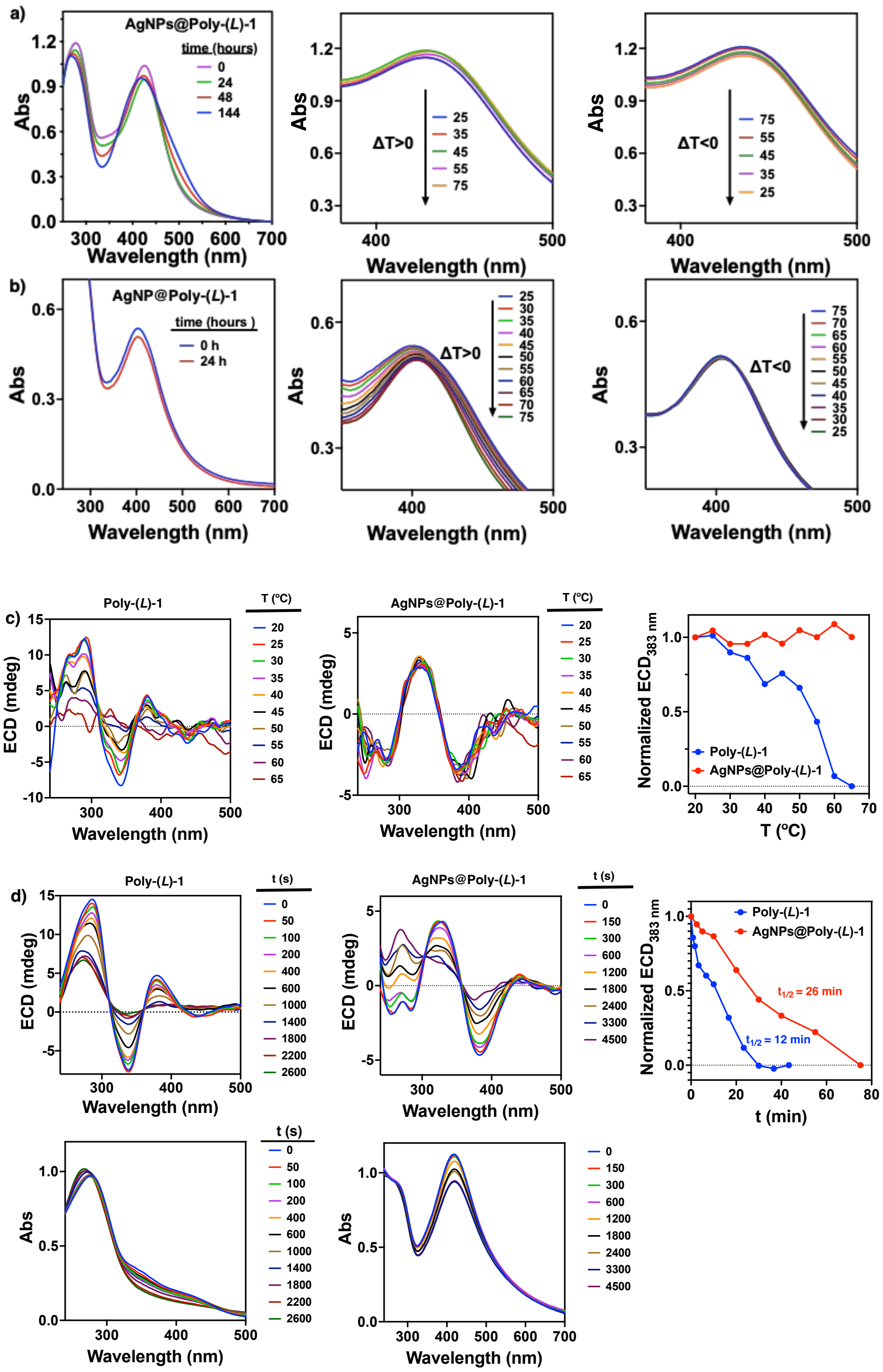


**Figure S12.** UV-Vis of (a) AgNPs@Poly-(*L*)-**1** prepared at pH=7 (b) AgNP@Poly-(*L*)-**1** prepared at pH=2 evolution by Time and Temperature (increase and decrease at 0.5 K $min^{-1}$).
(c) VT-ECD studies of Poly-(*L*)-**1** and AgNPs@Poly-(*L*)-**1** prepared at pH=7 (increase and decrease at 0.5 K $min^{-1}$). (d) ECD and UV-Vis spectra of Poly-(*L*)-**1** and AgNPs@Poly-(*L*)-**1** under light irradiation with time.

(d) ECD and UV-Vis studies of Poly-(*L*)-**1** and AgNPs@Poly-(*L*)-**1** prepared at pH=7 ) at different irradiation times under visible light.

## 10. ECD and UV-Vis studies of the oxidation of AgNP@Poly-(*L*)-1 with $Fe(ClO_4)_3$, $Hg(ClO_4)_2$ and $Ce(SO_4)_2$.

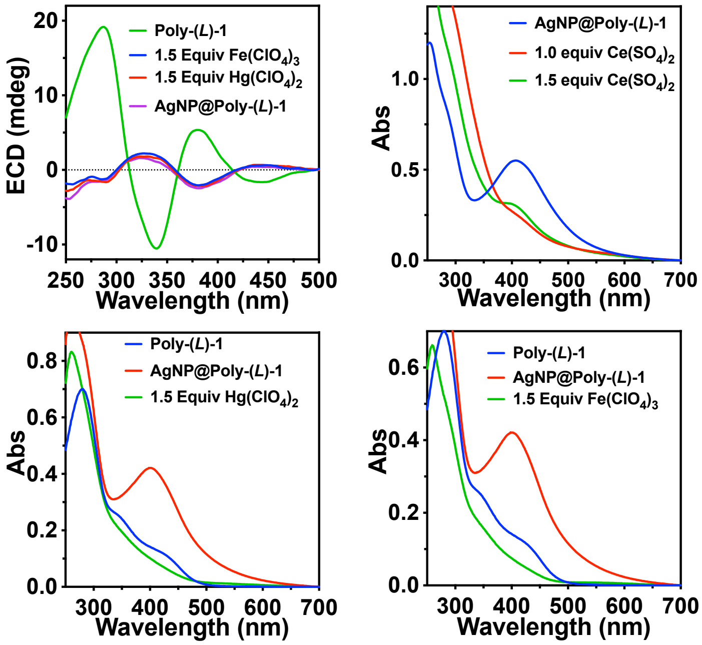


**Figure S13.** ECD and UV-Vis of AgNP@Poly-(*L*)-**1** in presence of $Fe(ClO_4)_3$, $Hg(ClO_4)_2$, $Ce(SO_4)_2$ (10 mg $mL^{-1}$ $H_2O$ MQ).

**11. ECD, UV-Vis and DLS studies of the oxidation of AgNPs@Poly-(*L*)-1 with $Fe(ClO_4)_3$, $Hg(ClO_4)_2$ and $Ce(SO_4)_2$.**

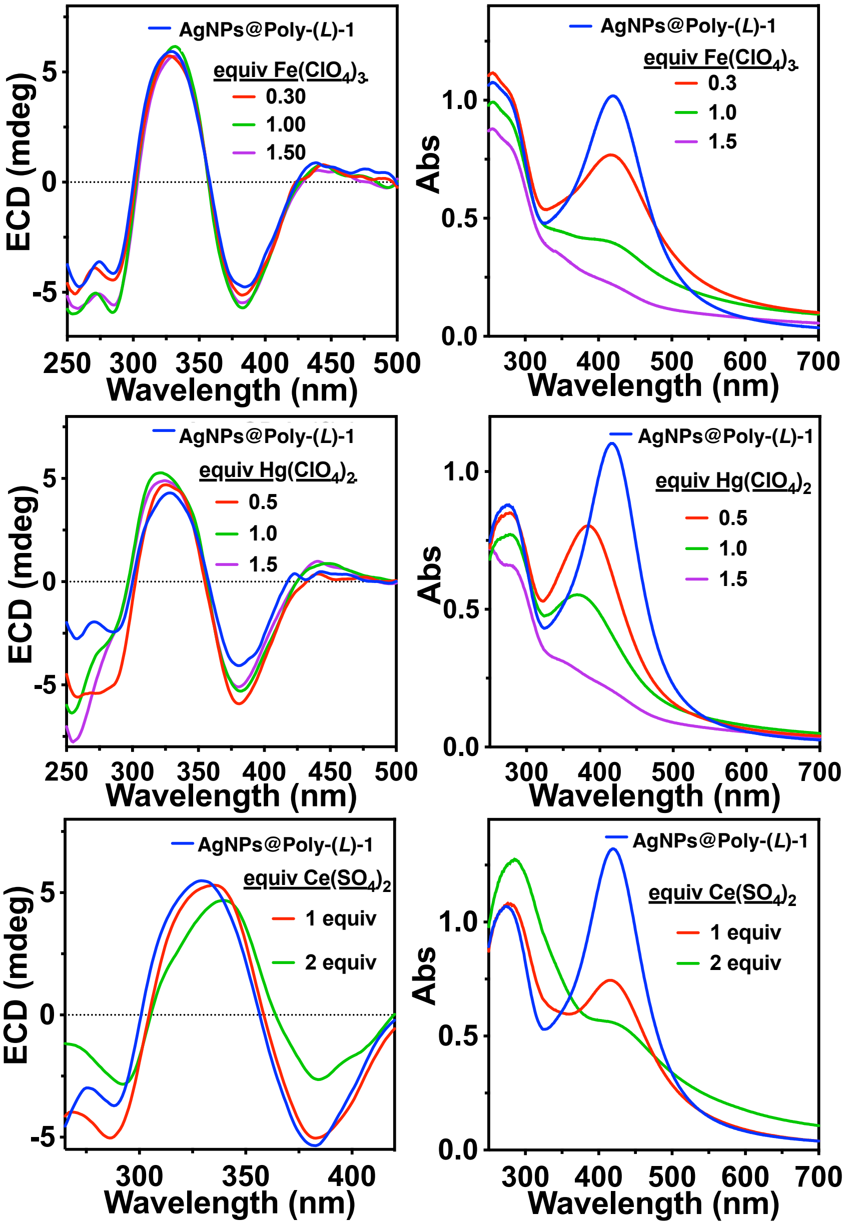


**Figure S14.** ECD and UV-Vis of AgNPs@Poly-(*L*)-**1** in presence of $Fe(ClO_4)_3$, $Hg(ClO_4)_2$, $Ce(SO_4)_2$ (10 mg $mL^{-1}$ $H_2O$ MQ).

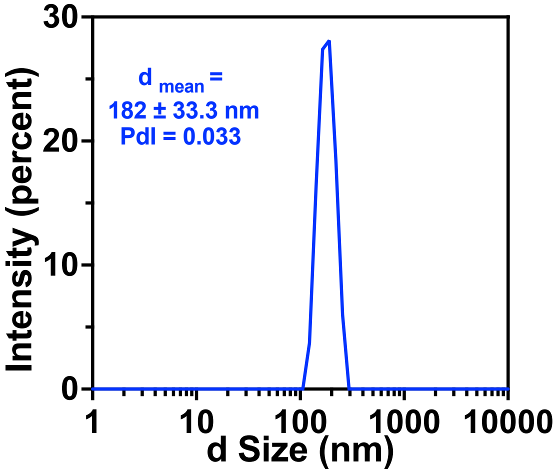


**Figure S15.** DLS of the obtained HPMC of AgNPs@Poly-(*L*)-**1** after the oxidation with $Fe(ClO_4)_3$ (10 mg $mL^{-1}$ $H_2O$ MQ).

## 12. ECD and UV-Vis studies of AgNPs@Poly-(*L*)-1 in the presence of ultrapure $FeCl_2$ and not ultrapure $Fe(ClO_4)_2$.

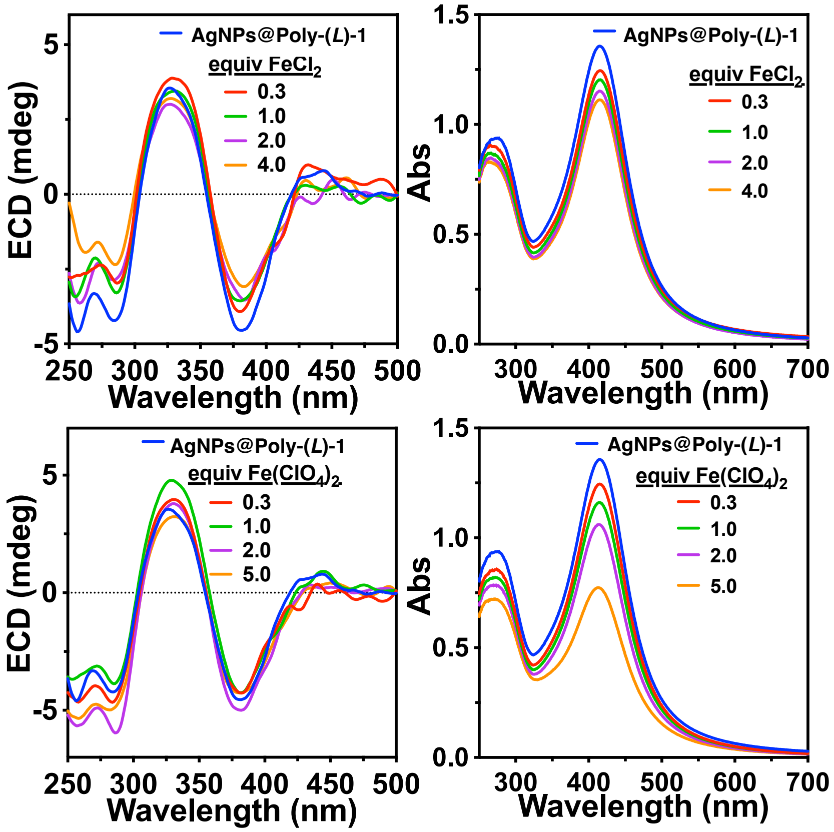


**Figure S16.** ECD and UV-Vis of AgNPs@Poly-(*L*)-**1** in presence of oxidated $Fe(ClO_4)_2$ and ultrapure $FeCl_2$ (10 mg $mL^{-1}$ $H_2O$ MQ).

## 13. ECD and UV-Vis studies of AgNPs@Poly-(*L*)-1 in the presence of salts.

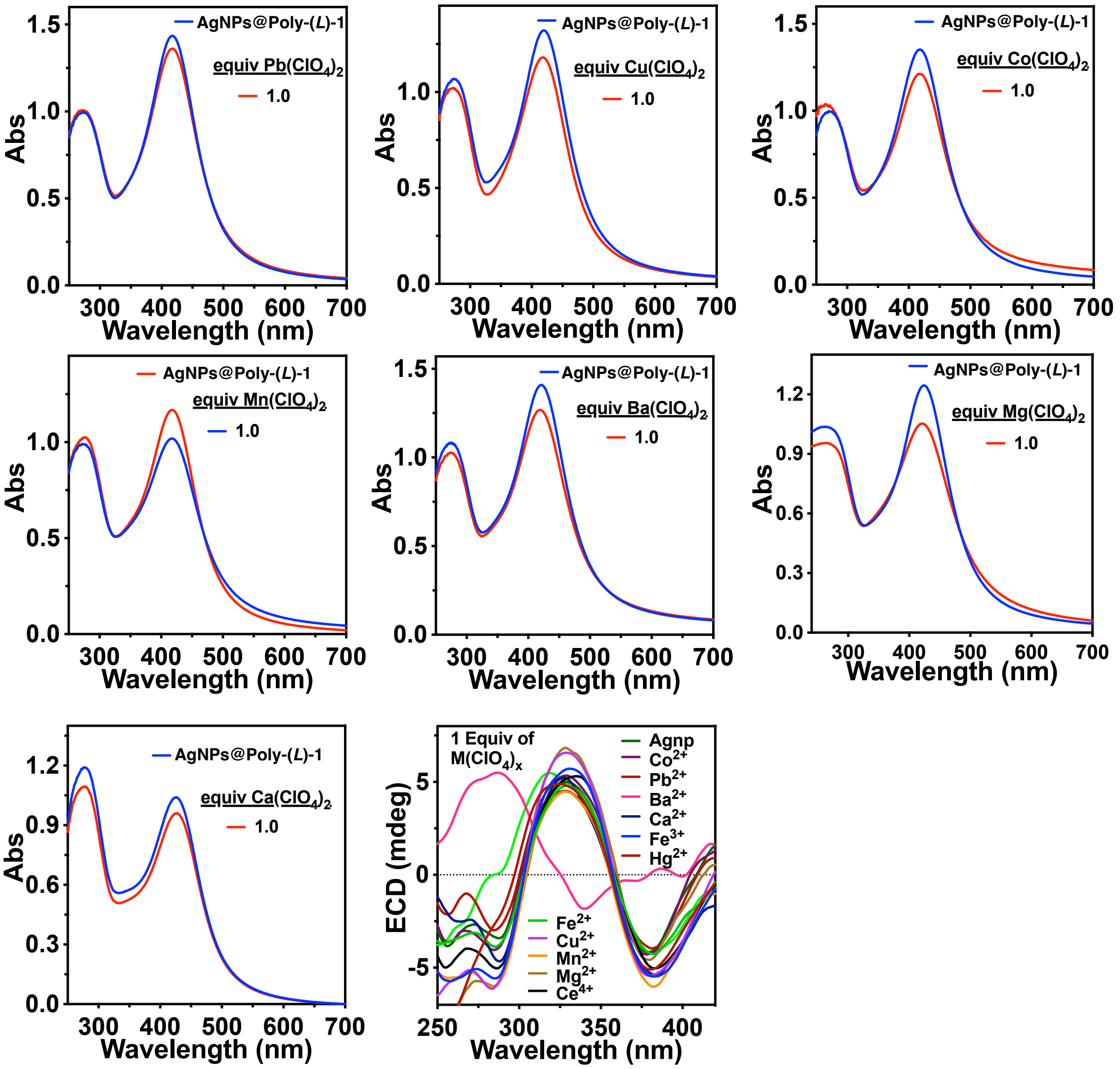


**Figure S17.** UV-Vis of AgNPs@Poly-(*L*)-**1** in presence of 1 equivalent of $M(ClO_4)_2$ salts (10 mg $mL^{-1}$ $H_2O$ MQ). ECD of AgNPs@Poly-(*L*)-**1** in presence of 1 equivalent of $M(ClO_4)_n$ and $Ce(SO_4)_2$ salts (10 mg $mL^{-1}$ $H_2O$ MQ).

## 14. ECD study of Poly-(*L*)-1 interaction with $Ba(ClO_4)_2$.

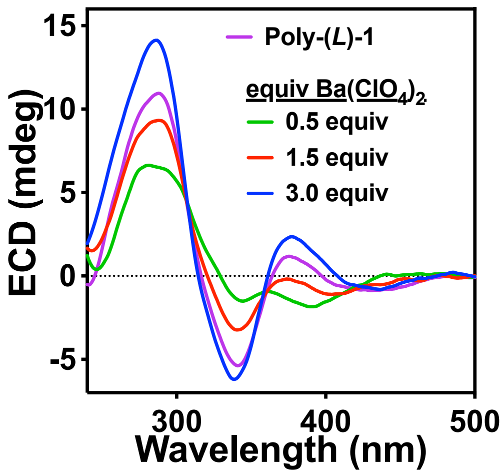


**Figure S18.** ECD of Poly-(*L*)-**1** in presence of $Ba(ClO_4)_2$ (10 mg $mL^{-1}$ $H_2O$ MQ).

## 15. Chiroptical study of AgNPs@Poly-(*L*)-1 and of AgNP@Poly-(*L*)-1 nanocomposites in the presence of divalent perchlorate salts.

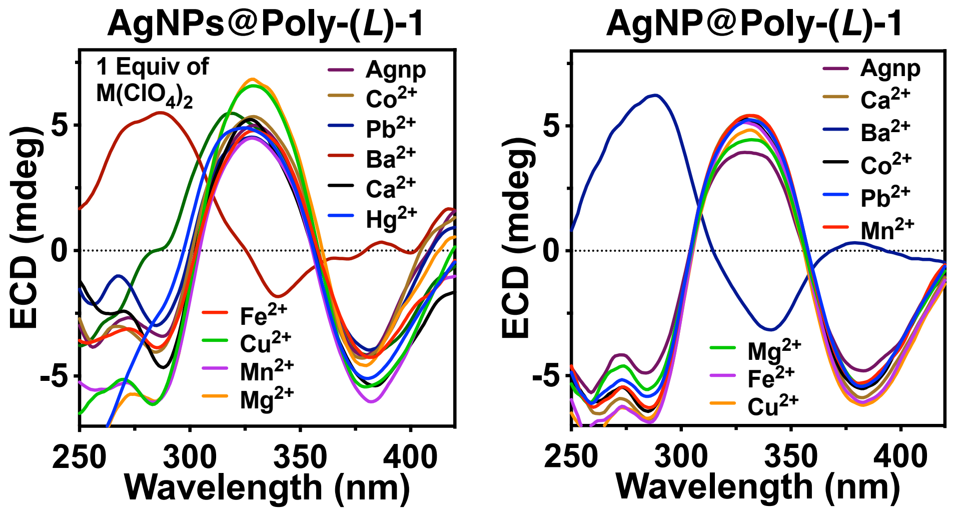


**Figure S19.** ECD of the nanocomposites AgNPs@Poly-(*L*)-**1** and AgNP@Poly-(*L*)-**1** in presence of $M(ClO_4)_2$ (10 mg $mL^{-1}$ $H_2O$ MQ).

## 16. UV-Vis of the nanocomposites after basification.

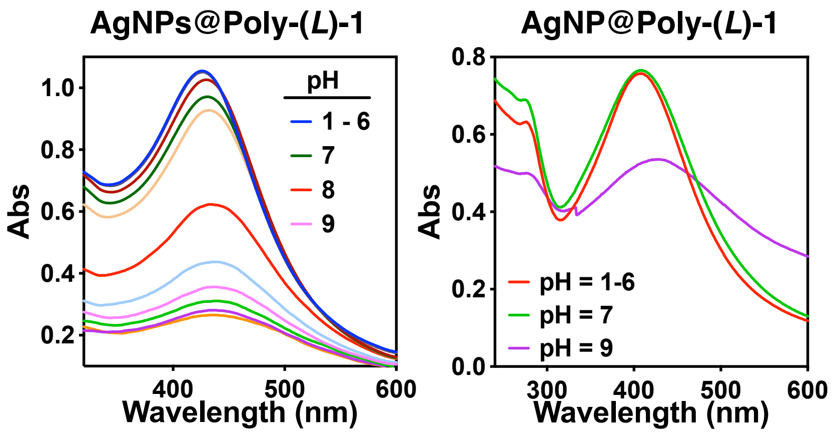


**Figure S20.** UV-Vis of the nanocomposites AgNPs@Poly-(*L*)-**1** and AgNP@Poly-(*L*)-**1** at different pH (basifying with NaOH 1M). NaOH 1M added in AgNP(s)@Poly-(*L*)-**1** with a microfluidic equipment.

## 17. UV-Vis and DLS of the nanocomposite following cycles of basification and acidification.

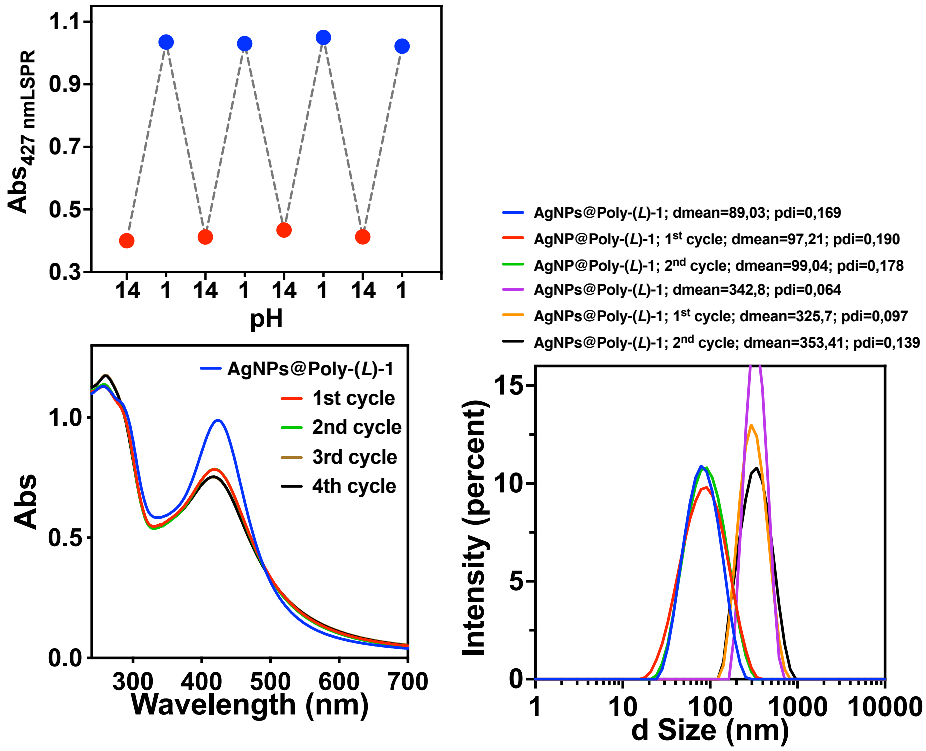


**Figure S21.** UV-Vis of nanocomposites AgNPs@Poly-(*L*)-**1** and DLS of the nanocomposites AgNP(s)@Poly-(*L*)-**1** after precipitation by basification with NaOH 1M followed by acidification with HCl 1M to pH = 1.

**18. Poly-(*L*)-1 at different pH.**

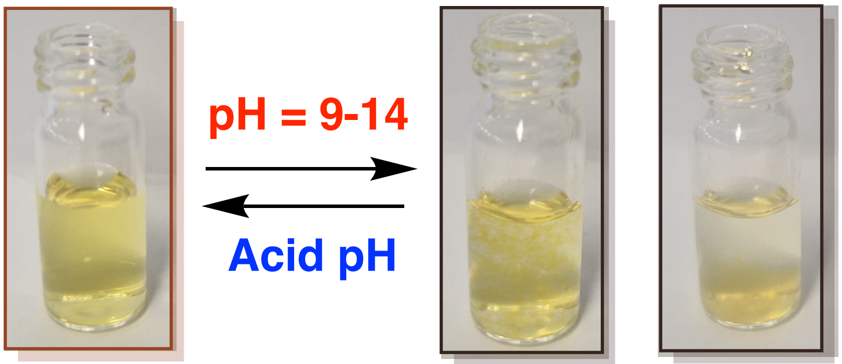


**Figure S22.** Precipitation of Poly-(*L*)-**1** at basic pH, and recovery after acidification.

**19. Scanning electron microscopy images of Poly-(*L*)-1/$AgClO_4$ pH = 7.**

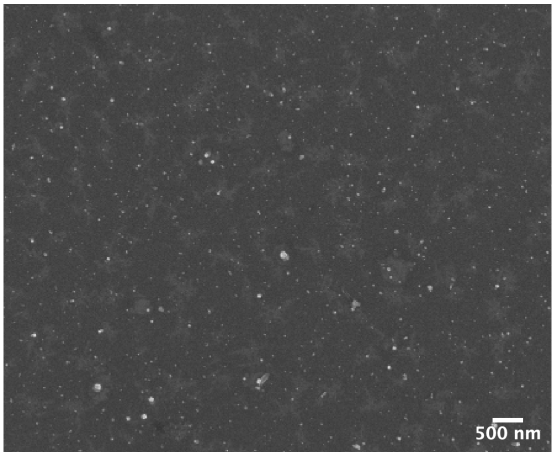


**Figure S23.** Scanning electron microscopy images of Poly-(*L*)-1/$AgClO_4$ (1/1.5 mol/mol) HPMC.

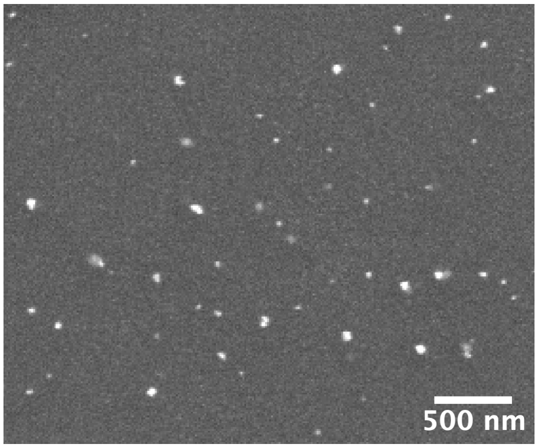


**Figure S24.** Scanning electron microscopy images of Poly-(*L*)-1/$AgClO_4$ (1/1.5 mol/mol) HPMC.

## 20. Scanning electron microscopy images of AgNP@Poly-(*L*)-1 synthetized at pH = 2.

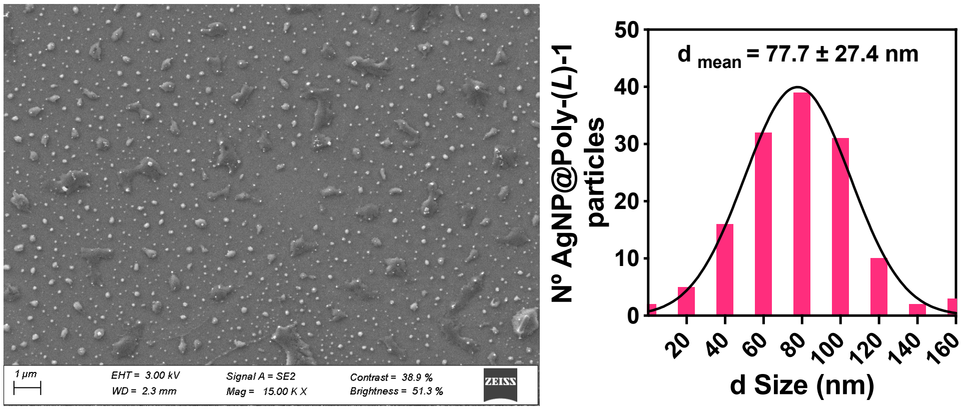


**Figure S25.** Scanning electron microscopy images of AgNP@Poly-(*L*)-1 nanocomposite used for the gaussian size distribution nanocomposites synthetized at pH = 2 (140 nanocomposites measured).

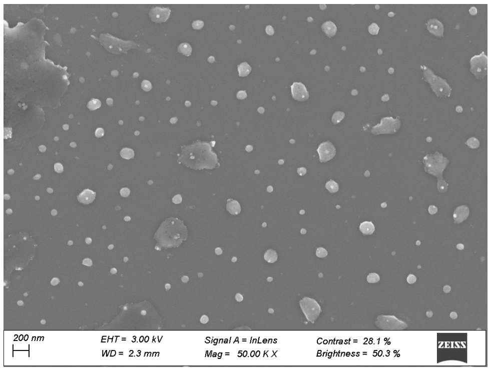


**Figure S26.** Scanning electron microscopy images of AgNP@Poly-(*L*)-1 nanocomposite synthetized at pH = 2.

## 21. Transmission electron microscopy images of AgNP@ Poly-(*L*)-1 synthetized at pH = 2.

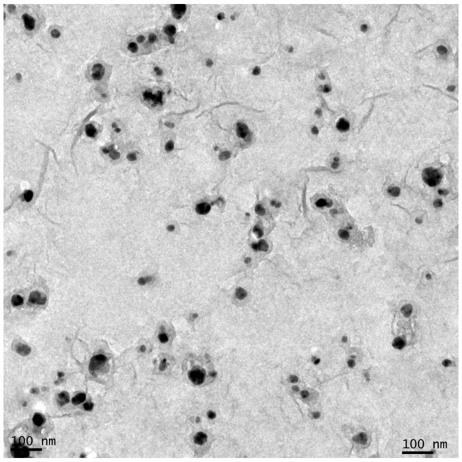


**Figure S27.** Transmission electron microscopy images of AgNP@Poly-(*L*)-**1** nanocomposite synthetized at pH = 2 used for the gaussian size distribution of the AgNPs (50 AgNPs measured).

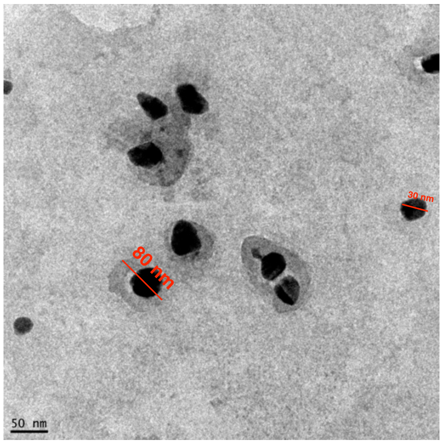


**Figure S28.** Transmission electron microscopy images of AgNP@Poly-(*L*)-**1** nanocomposite synthetized at pH = 2.

## 22. Scanning electron microscopy images of AgNPs@ Poly-(*L*)-1 synthetized at pH = 7.

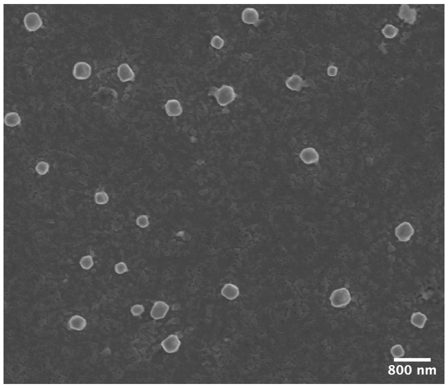


**Figure S29.** Scanning electron microscopy images of AgNPs@Poly-(*L*)-1 nanocomposite synthetized at pH = 7.

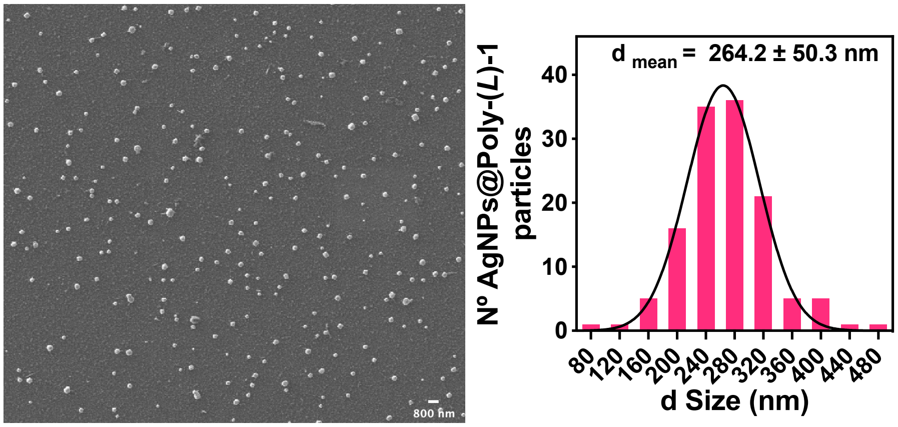


**Figure S30.** Scanning electron microscopy images of AgNPs@Poly-(*L*)-1 nanocomposite synthetized at pH = 7 used for the gaussian size distribution of the nanocomposites (140 nanocomposites measured).

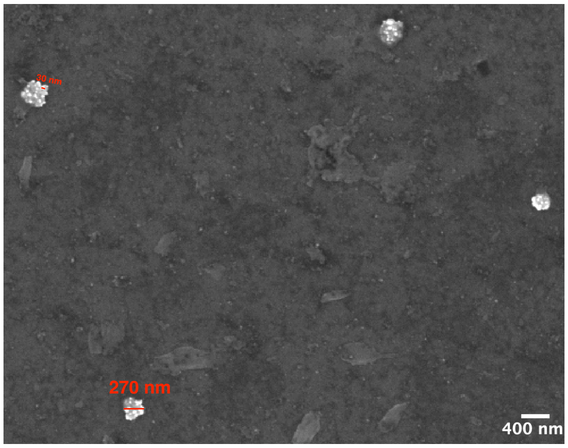


**Figure S31.** Scanning electron microscopy images of AgNPs@Poly-(*L*)-1 nanocomposite synthetized at pH = 7.

## 23. Transmission electron microscopy images of AgNP@ Poly-(*L*)-1 synthetized at pH = 7.

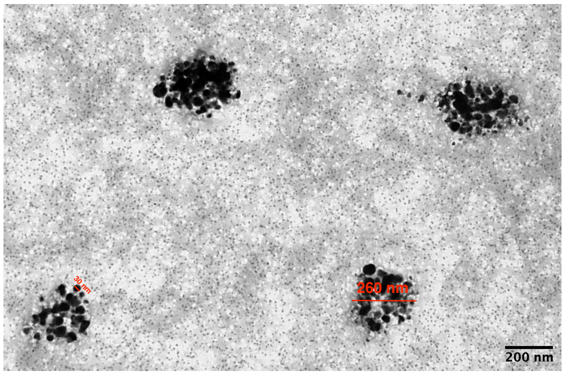


**Figure S32.** Transmission electron microscopy images of AgNPS@Poly-(*L*)-**1** nanocomposite synthetized at pH = 7 used for the gaussian size distribution of the AgNPs (100 AgNPs measured).

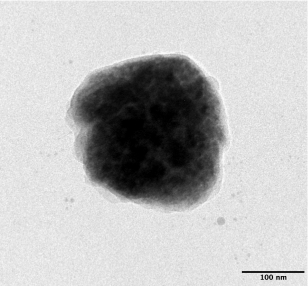

**Figure S33.** Transmission electron microscopy images of AgNPs@Poly-(*L*)-**1** nanocomposite synthetized at pH = 7.

**24. Scanning electron microscopy images of AgNPs@ Poly-(*L*)-1 synthetized at pH = 7 before after precipitation with NaOH 1M and recovery with HCl 1M**

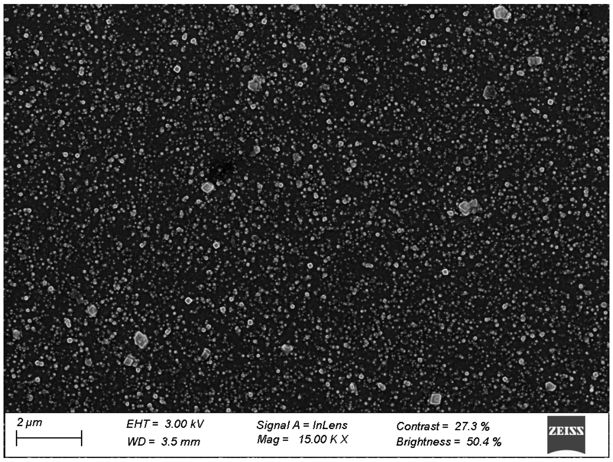


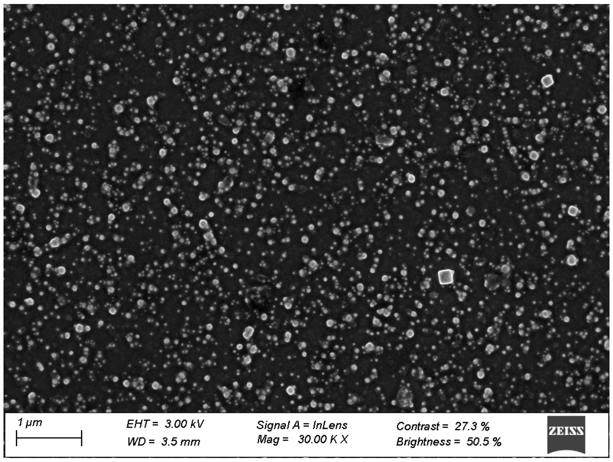


**Figure S34.** Scanning electron microscopy images of AgNPs@Poly-(*L*)-1 nanocomposite synthetized at pH = 7 before precipitation with NaOH 1M.

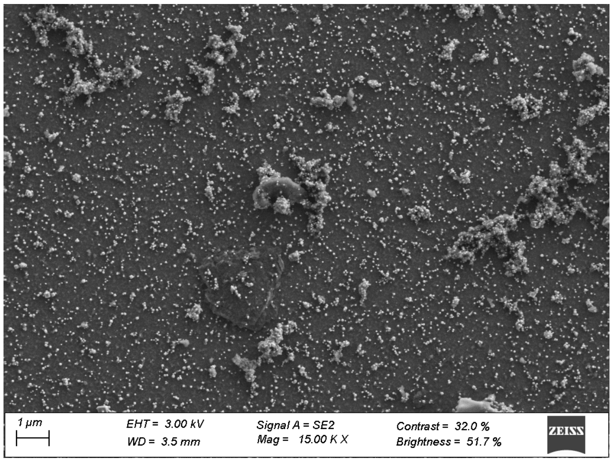


**Figure S35.** Scanning electron microscopy images of AgNPs@Poly-(*L*)-1 nanocomposite synthetized at pH = 7 after precipitation with NaOH 1M and redispersion with HCl 1M two times.

**25. Scanning electron microscopy images of AgNP@ Poly-(*L*)-1 synthetized at pH = 2 before after precipitation with NaOH 1M and recovery with HCl 1M**

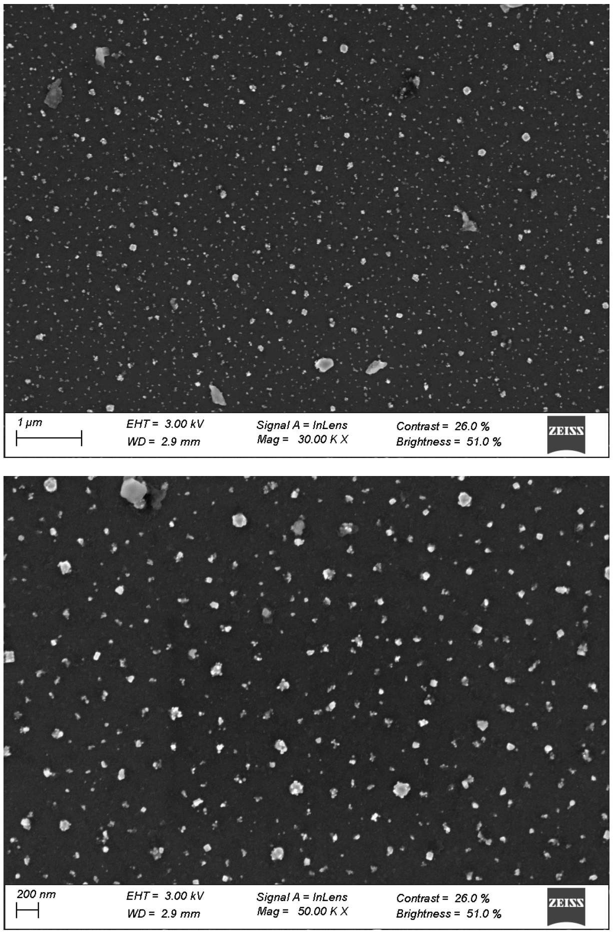


**Figure S36.** Scanning electron microscopy images of AgNP@Poly-(*L*)-1 nanocomposite synthetized at pH = 2 before precipitation with NaOH 1M.

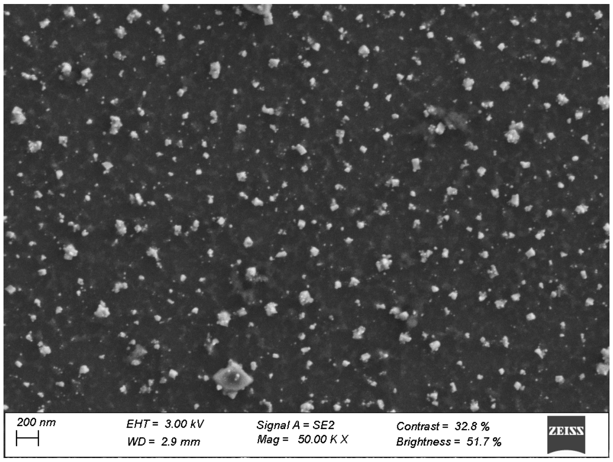


**Figure S37.** Scanning electron microscopy images of AgNP@Poly-(*L*)-1 nanocomposite synthetized at pH = 2 after precipitation with NaOH 1M and redispersion with HCl 1M two times.

**26. UV-Vis and TEM studies of AgNP@Poly-(*L*)-1 nanocomposite prepared (a) at pH = 7 and measured at pH = 2 (b) prepared at pH = 2 and measured at pH = 7**

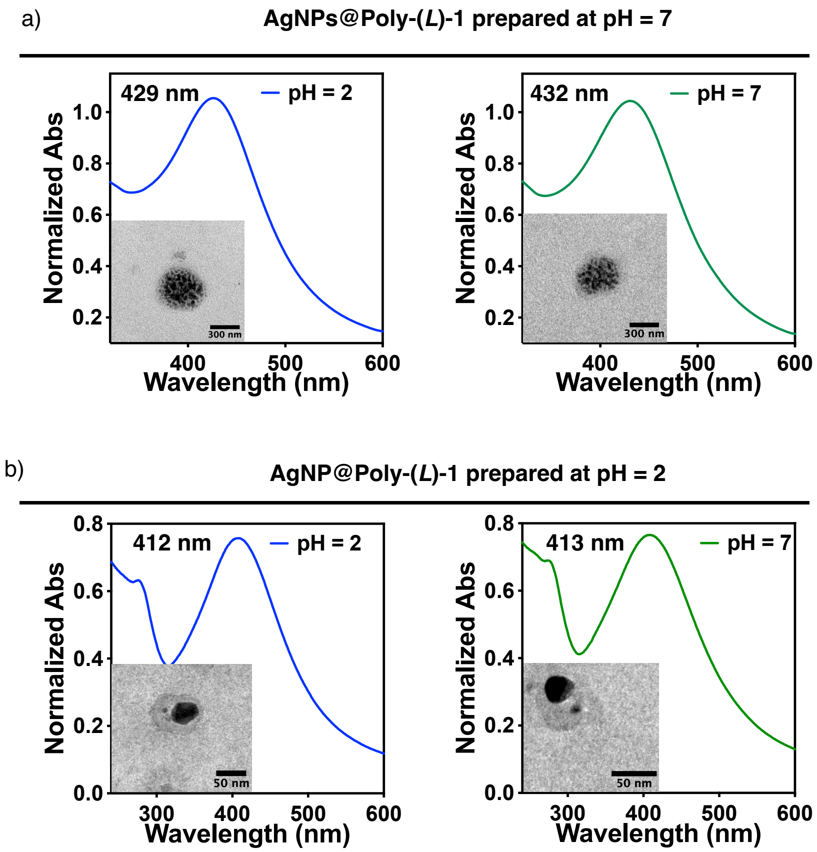


**Figure S38.** UV-Vis spectra and TEM images of AgNP@Poly-(*L*)-1 nanocomposite (a) synthetized at pH = 7 and measured at pH = 2 and (b) prepared at pH = 2 and measured at pH = 7.

## 27. Theoretical Calculations

To confirm the mechanism described, DFT calculations were performed with Gaussian 16 Rec.01c. [S2] The optimization and frequency calculations were performed in the monomer (*S*)-**1** using the method set B3LYP [S3] with a modification to add a grimme Dispersion 3 [S4] and the basis set 6-311+g(d,p) [S5] for H,C,O and N and cc-pVDZ-PP [S6] for silver [S7] obtained from Basis Set Exchange (BSE) [S8] in a polarizable continuum model (PCM) of water. The Free energies (G) of (*L*)-**1**, (*L*)-**1**/$Ag^+$ and (*L*)-**1**/$Ag^0_7$ with different conformations are compilated in the Table S2. The possible coordination mechanisms or pathways with the Free energy difference are represented in Figures s39 and s42. The interaction with silver cation and $Ag^0_7$ that represents the facet of AgNP was followed by the Electron localized Field (ELF) and Non-Covalent Interaction (NCI) analysis of the checkfile from gaussian using Multiwfn [S9]. The NCI represents with blue color the strong interactions, red colour for repulsion and green colour for VdW (Figures S40 and S41).

**Table S2.** Free energies of the optimized molecules used to calculate the Free Energy difference (B3LYP-D3 with 6-311+g(d,p) for H,C,O and N and cc-pVDZ-PP for silver). The most stable structures are highlighted in red.

| Compound | G (kcal/mol) |
|---|---|
| $Ag^+$ | 92150.26754 |
| $Cl^-$ | 288919.7102 |
| $Ag^0_7$ (111) | 645811.8223 |
| (*L*)-1-$NH_2$ *ap* | -457461.8779 |
| (*L*)-1-$NH_2$ *sp* | -457457.6724 |
| (*L*)-1-$NH_3^+Cl^-$ *ap* | -746649,5965 |
| (*L*)-1-$NH_3^+Cl^-$ *sp* | -746510,1798 |
| (*L*)-1-$NH_2$ *sp* BiChelate C=O···$Ag^+$···$NH_2$ | -549622.0129 |
| (*L*)-1-$NH_2$ *sp* MonoChelate O=C—HN···$Ag^+$ | --549605,0403 |
| (*L*)-1-$NH_2$ *ap* BiChelate $H_2N$···$Ag^+$···NH—C=O | -549619,9032 |
| (*L*)-1-$NH_2$ *ap* MonoChelate C=O ···$Ag^+$ | -549617,2815 |
| (*L*)-1-$NH_3^+$···$Ag^0_7$ *sp* | -1103555,133 |
| (*L*)-1-$NH_3^+$···$Ag^0_7$ *ap* | -1103549,017 |
| (*L*)-1-$NH_2$···$Ag^0_7$ *sp* | -1103278,29 |
| (*L*)-1-$NH_2$···$Ag^0_7$ *ap* | -1103280,638 |

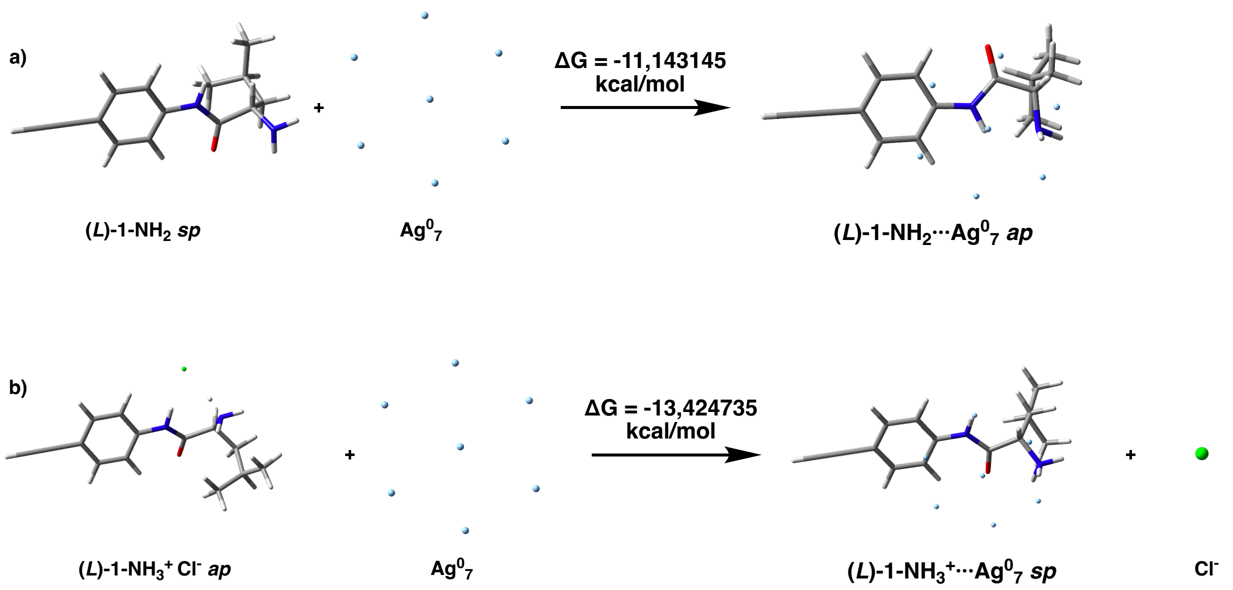


**Figure S39.** Free Energy difference and optimized geometries (B3LYP-D3 with 6-311+g(d,p) for H,C,O and N and cc-pVDZ-PP for silver) of (a) (*L*)-1-$NH_2$ in *sp* conformation, $Ag^0_7$ and (*L*)-1-$NH_2$---$Ag^0_7$ in *ap* conformation (b) (*S*)-1-$NH_3^+Cl^-$ in *ap* conformation, $Ag^0_7$, (*L*)-1-$NH_3^+$---$Ag^0_7$ in *ap* conformation and $Cl^-$.

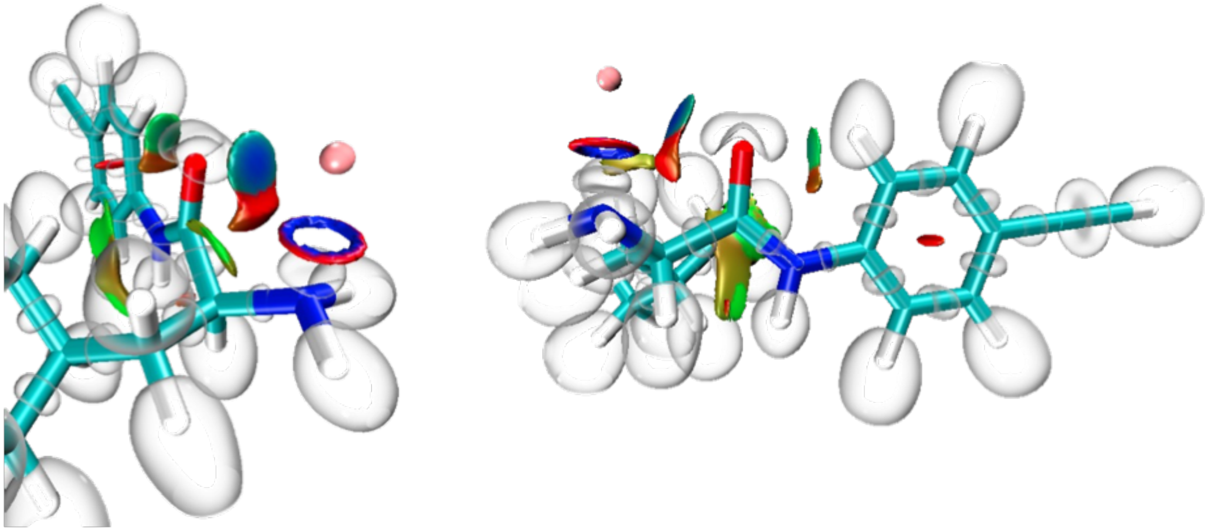

**Figure S40.** NCI and ELF representation of the Ag cation interacting with the carbonyl and the free electron pair of $NH_2$ in (*L*)-1-$NH_2$ in *sp* conformation.

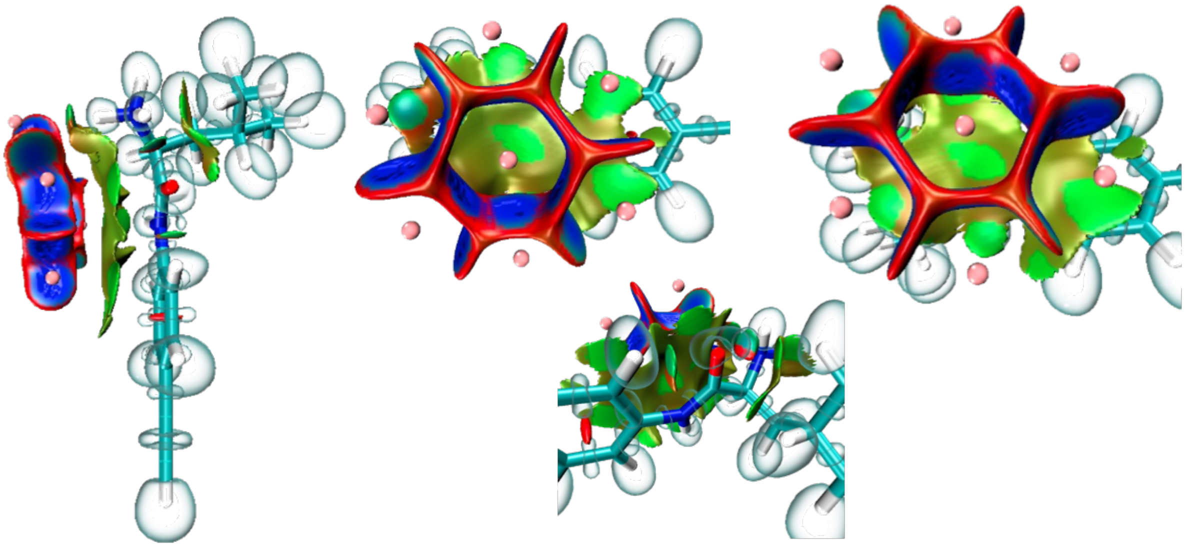

**Figure S41.** NCI and ELF representation of the $Ag^0_7$ facet of a AgNP interacting with the carbonyl and a proton of $NH_3^+$ in (*L*)-1-$NH_3^+$ in *sp* conformation.

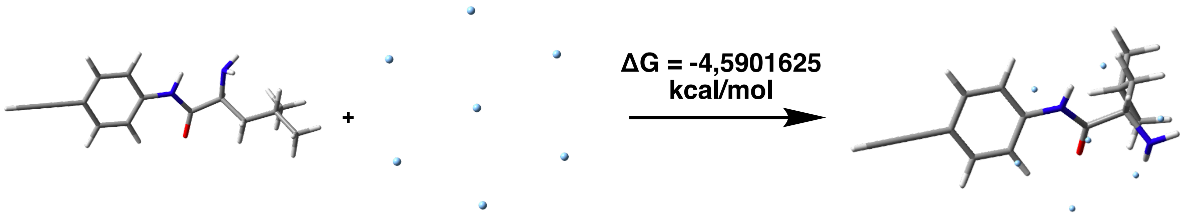


**(*L*)-1-$NH_2$ *ap*** **$Ag^0_7$** **(*L*)-1-$NH_2 \cdots Ag^0_7$ *sp***

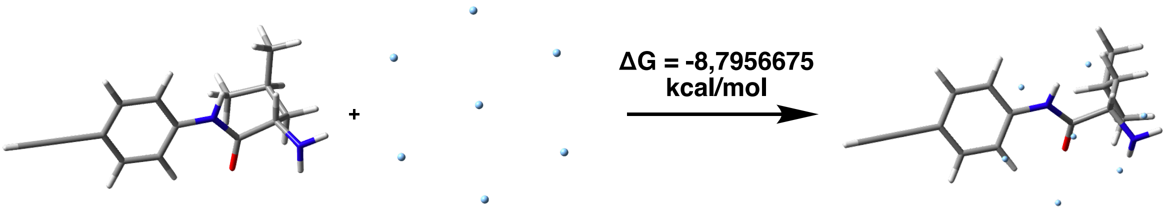


**(*L*)-1-$NH_2$ *sp*** **$Ag^0_7$** **(*L*)-1-$NH_2 \cdots Ag^0_7$ *sp***

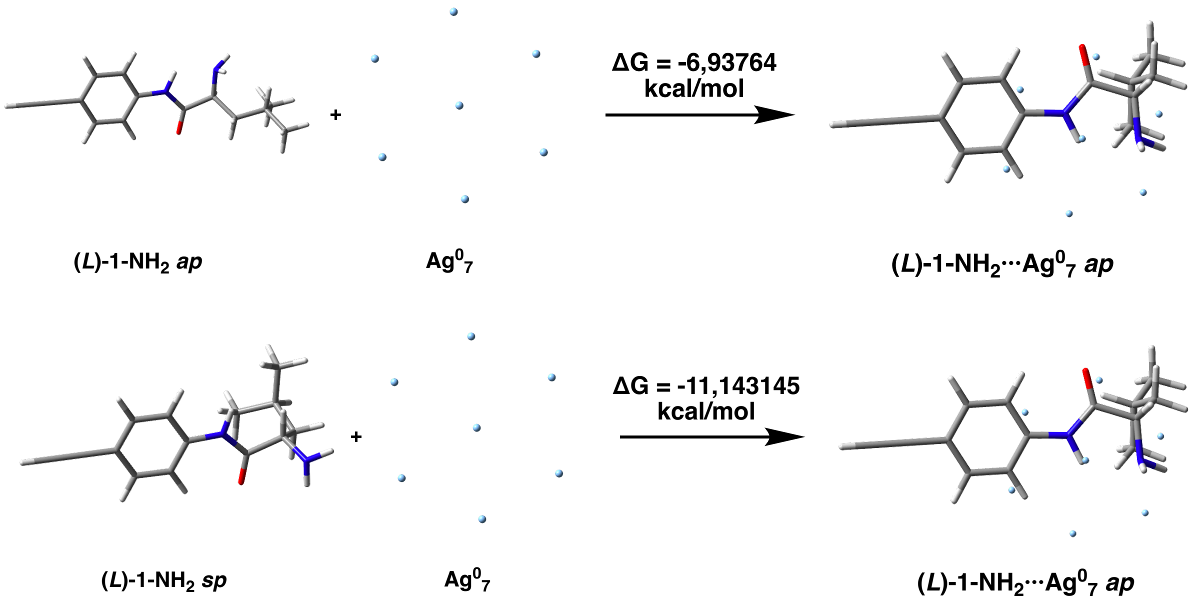


**Figure S42.** Optimized geometries (B3LYP-D3 with 6-311+g(d,p) for H,C,O and N and cc-pVDZ-PP for silver) and alternative proposed mechanisms that are less likely to occur due to smaller Free energy difference.

## 28. References


[S1] E. Suárez-Picado, E. Quiñoá, R. Riguera, F. Freire, Poly(phenylacetylene) Amines: A General Route to Water-Soluble Helical Polyamines, *Chem. Mater.* **2018**, *30*, 6908–6914.

[S2] Gaussian 16, Revision C.01, M. J. Frisch, G. W. Trucks, H. B. Schlegel, G. E. Scuseria, M. A. Robb, J. R. Cheeseman, G. Scalmani, V. Barone, G. A. Petersson, H. Nakatsuji, X. Li, M. Caricato, A. V. Marenich, J. Bloino, B. G. Janesko, R. Gomperts, B. Mennucci, H. P. Hratchian, J. V. Ortiz, A. F. Izmaylov, J. L. Sonnenberg, D. Williams-Young, F. Ding, F. Lipparini, F. Egidi, J. Goings, B. Peng, A. Petrone, T. Henderson, D. Ranasinghe, V. G. Zakrzewski, J. Gao, N. Rega, G. Zheng, W. Liang, M. Hada, M. Ehara, K. Toyota, R. Fukuda, J. Hasegawa, M. Ishida, T. Nakajima, Y. Honda, O. Kitao, H. Nakai, T. Vreven, K. Throssell, J. A. Montgomery, Jr., J. E. Peralta, F. Ogliaro, M. J. Bearpark, J. J. Heyd, E. N. Brothers, K. N. Kudin, V. N. Staroverov, T. A. Keith, R. Kobayashi, J. Normand, K. Raghavachari, A. P. Rendell, J. C. Burant, S. S. Iyengar, J. Tomasi, M. Cossi, J. M. Millam, M. Klene, C. Adamo, R. Cammi, J. W. Ochterski, R. L. Martin, K. Morokuma, O. Farkas, J. B. Foresman, and D. J. Fox, Gaussian, Inc., Wallingford CT, **2016**.

[S3] A. D. Becke, Density-functional thermochemistry. III. The role of exact exchange**,** *The Journal of Chemical Physics*, **1993**, *98*, 5648–5652.

[S4] S. Grimme, J. Antony, S. Ehrlich, H. Krieg, A consistent and accurate *ab initio* parametrization of density functional dispersion correction (DFT-D) for the 94 elements H-Pu, *The Journal of Chemical Physics* **2010**, *132,* 154104

[S5] V. A. Rassolov, M. A. Ratner, J. A. Pople, P. C. Redfern, L. A. Curtiss, 6-31G* basis set for third-row atoms, *J. Comput. Chem.*, **2001**, *22*, 976–984.

[S6] D. Figgen, G. Rauhut, M. Dolg, H. Stoll, Energy-consistent pseudopotentials for group 11 and 12 atoms: adjustment to multi-configuration Dirac–Hartree–Fock data, *Chemical Physics* **2005**, *311*, 227–244.

[S7] K. A. Peterson, C. Puzzarini, Systematically convergent basis sets for transition metals. II. Pseudopotential-based correlation consistent basis sets for the group 11 (Cu, Ag, Au) and 12 (Zn, Cd, Hg) elements, *Theor Chem Acc* **2005**, *114*, 283–296.

[S8] B. P. Pritchard, D. Altarawy, B. Didier, T. D. Gibson, T. L. Windus, New Basis Set Exchange: An Open, Up-to-Date Resource for the Molecular Sciences Community, *J. Chem. Inf. Model.* **2019**, *59*, 4814–4820.

[S9] T. Lu, F. Chen, Multiwfn: A multifunctional wavefunction analyzer, *J Comput Chem* **2011**, *33*, 580–592.
